# Data-driven scenario analysis supports the revival of historic silvoarable systems for carbon smart rural landscapes

**DOI:** 10.1038/s41598-025-18950-7

**Published:** 2025-10-07

**Authors:** Filippo Brandolini, Angelo Gurgel, Andrea Zerboni

**Affiliations:** 1 Center for Sustainability Science and Strategy, Massachusetts Institute of Technology, 400 Main St, Cambridge, MA 02142 USA; 2 Dipartimento di Scienze della Terra “Ardito Desio”, Università degli Studi di Milano, Via Luigi Mangiagalli 34, 20133 Milan, Italy

**Keywords:** Archaeology, Climate-change adaptation, Ecological modelling

## Abstract

Agroforestry has long been recognised as a nature-based solution for climate mitigation, yet its adoption in Europe has drastically declined due to the socio-economic transformations and land use intensification since the onset of the *Great Acceleration* (ca. mid-twentieth century). This study reconstructs the historical role of agroforestry in Northern Italy by drawing on century-long land use records (1929–2024) and historical sources, which were crucial for identifying and modelling the carbon stock of traditional silvoarable systems. Through the integration of Monte Carlo simulations and scenario-based modelling, we estimate that historic silvoarable systems stored an average of 75.4 t C ha^−1^, with a potential range of 50.4–101.6 t C ha^−1^. The widespread abandonment of agroforestry practices led to a 97% reduction in their extent, accompanied by a corresponding expansion of monocultures. Future management scenarios suggest that restoring silvoarable systems could enhance regional carbon sequestration by up to 12%, a gain comparable to afforestation strategies requiring the conversion of 25% of existing farmland. Our findings underscore the global value of traditional ecological knowledge and historical land use strategies in informing carbon-smart agricultural transitions and shaping policies for resilient, multifunctional landscapes.

**Supplementary Information:**

The online version contains supplementary material available at 10.1038/s41598-025-18950-7.

## Introduction

Carbon dioxide (CO_2_) is one of the major greenhouse gases (GHGs) present in the atmosphere, and its capture, storage, and sequestration are key components of the mitigation pathways outlined by the Intergovernmental Panel on Climate Change^1^. Land use (LU) strategies play a crucial role in global CO_2_ cycles, and the Paris Agreement underscores the importance of sustainable land management in global climate mitigation efforts^2^. Agriculture represents the largest anthropogenic LU globally^3^ and contributes between 18 and 24% of GHG emissions^4^, with CO_2_ accounting for 27% of this footprint^5^. While conservation initiatives have led to a 10% reduction in global agricultural CO_2_ emissions over the past decade, further reductions are necessary to meet climate targets^6^. Agroforestry (AF) (i.e., the integration of trees with crops or livestock) is recognised as one of the most promising nature-based carbon smart solutions within regenerative agriculture. It not only mitigates emissions^7^ but also enhances carbon storage, with a global potential ranging from 0.12 to 0.31 Pg C y^−1^^8^, while simultaneously providing a wide range of environmental benefits (e.g. soil health improvement, erosion control, water regulation, biodiversity conservation, air quality enhancement, microclimate regulation, pollination support, pest and disease control)^9,10^. AF is now acknowledged not only as a Nature-based Climate Solution, but also as a Natural Removal Solution, due to its capacity to deliver durable and verifiable carbon sequestration^11^. It has recently gained formal recognition within voluntary carbon markets, which offer financial incentives through certified credits for land-based carbon removals^12^. In this context, Regulation (EU) 2024/3012 establishes a Union certification framework for permanent carbon removals and explicitly lists AF among the eligible carbon farming practices, thereby paving the way for its broader integration into both climate policy and market-based mechanisms^13^.

Silvoarable (SA) and silvopastoral (SP) systems represent the two main subtypes of AF. SA systems consist of widely spaced trees intercropped with annual or perennial crops, including alley cropping, scattered trees, and hedgerows. In contrast, SP systems combine trees with forage and animal production, encompassing both forest or woodland grazing and the use of open forest trees, where livestock benefit from the forage provided beneath the canopy while contributing to landscape management^14,15^.

When considering the Old World and the archaeological and historical roots of present-day AF, it is noteworthy that AF played a central role in shaping European farmland—particularly from the Late Middle Ages (c. 1300–1500 CE)— and contributed to the development of a diverse range of multifunctional historic landscapes^16^. These include both SA and SP systems, such as the *Bocage* and *Joualle* systems in France^17^, hedgerows in Belgium^18^, the *Dehesa* in Spain^19,20^, the *Montado* in Portugal^21^, *Streuobst* in Germany^22^, *Plužiny* in the Czech Republic^23,24^, *Prinones* in Greece^25^, and *Coltura Promiscua* in Italy^26^. Today, agriculture occupies approximately 39% of the European Union (EU) total land area (157 Mha)^27^ and remains a significant source of GHG emissions, accounting for around 12% of the EU’s total emissions^28^. In the same region, AF now occupies just 9% of the EU’s rural land, primarily in the form of SP systems that have been preserved in the Mediterranean areas, particularly in the Iberian Peninsula and Greece^27^. The sharp decline of SA systems was largely driven by the socio-economic and LU and land cover (LULC) transformations associated with the *Great Acceleration*, a period of rapid industrialisation and agricultural intensification that began in the mid-twentieth century^29,30^. Considering this long-term decline and their environmental consequences, attention has increasingly shifted toward reconciling productivity with sustainability in modern agricultural systems, which is one of the most pressing challenges for both current and future rural development^31^. In this regard, SA systems offer a promising nature-based solution to enhance carbon sequestration, diversify yields and preserve cultural landscapes. Realizing this potential requires supportive policies, clear definitions, and further research into optimal designs and mechanization^32^. In this context, the EU recognises cultural heritage and traditional ecological knowledge (TEK) as a potential driver for enhancing rural well-being and fostering long-term socio-economic development^33^. Strategies to reduce CO_2_ emissions in agriculture focus on expanding forest conservation and implementing climate-smart practices that enhance carbon sequestration^34^. Through the Common Agricultural Policy (CAP) 2024–2027, the EU underscores the importance of nature-based solutions in transforming agriculture into a climate-resilient, carbon-sequestering sector^28^. A substantial share (32%) of the CAP budget is dedicated to environmental objectives, including financial support for AF practices that facilitate the establishment or restoration of traditional SA systems^28^. Despite these economic incentives, farmers across the EU remain sceptical about adopting AF (especially SA systems) due to a combination of technical, economic, and knowledge-related barriers. The major concerns include:

A. Technical challenges: a major impediment towards the adoption of SA systems is the lack of knowledge regarding the best combinations of trees, and crops suitable for specific regional conditions^35^.
B. Knowledge and awareness gaps: SA-AF is often regarded as an innovative system, yet traditional knowledge surrounding its implementation has declined due to agricultural intensification and mechanisation during the twentieth century CE^16,30,36^. This has led to a disconnect between historical land management practices and modern farming techniques^8,37^.
C. Limited availability of EU regional-scale models: the current SA-AF research remains heavily concentrated in tropical regions^38–41^. In contrast, studies in temperate European regions are limited^36,42,43^ and often rely on small-scale, single-farm experiments^44–48^. More regional-scale case studies are needed to showcase SA-AF’s viability under varying socio-economic and environmental conditions^8,37,49^.
D. Economic uncertainty: farmers often perceive SA systems as less productive than conventional monocultures, with uncertain short-, medium-, and long-term economic returns. This perception is particularly relevant in regions where these systems have largely disappeared and where practical demonstrations of economic viability are lacking^35,42^. Additionally, the inadequate demonstration of ecosystem service payments at the regional level (such as those for carbon sequestration) fails to provide sufficient economic motivation, as their long-term benefits remain uncertain^50^. To support the practical implementation of SA-AF, it is crucial to develop regionally adapted models that assess long-term ecosystem services and sustainability beyond the constraints of short-term experiments^35^.

Aside from farmers’ economic concerns, these barriers can be attributed to the erosion of TEK related to large-scale SA systems in the EU. Addressing this loss requires interdisciplinary approaches that reconnect historical LU systems with contemporary sustainability goals^51^. The integration of ecosystem service science, environmental history and landscape archaeology offers a powerful framework: historical and archaeological records inform ecosystem service science assessments by revealing patterns of land management in the past, while ecosystem service frameworks help quantify the sustainability of traditional practices^52–54^. Together, they promote a dynamic view of landscapes and support more holistic, transdisciplinary approaches to sustainable planning^55^.

Focusing on Northern Italy (Fig. 5) — the Po-Venetian Plain (PVP) is one of the most affected regions in EU in terms of atmospheric GHG concentrations (Supplementary Material - A) —this study examines the long-term impact of abandonment of historic SA systems on regional nature-based carbon stock (CS) throughout the twentieth century. The underlying hypothesis is that the abandonment of SA systems led to a general decrease in the CS capacity of the rural landscape, primarily due to the widespread removal of trees and the simplification of LU patterns, which reduced the overall biomass and soil organic carbon associated with AF mosaics. In addition to quantifying these dynamics, this case study serves to develop a protocol and test its reliability, reconnecting present day seek for sustainability to traditional LU practice potentially deeply rooted in the historical and archaeological records. To address challenges beyond the adoption and restoration of traditional SA systems in the EU, this research employs a scenario-based modelling approach, starting from historical observations from the pre-satellite era (< 1970s) combined with the most recent LULC dataset available. Drawing on historical records of the rural landscape from the 1920s, this research aims to:

- Quantify the loss of traditional SA systems during the Great Acceleration in the region;
- Track the influence of LULC changes on natural CS dynamics;
- Estimate the carbon sequestration potential of one of EU’s oldest SA systems (*Coltura Promiscua*), now entirely abandoned;
- Provide a realistic, regional-scale example of SA implementation, addressing the lack of long-term and large-scale demonstration models by utilising historical data;
- Simulate the potential benefits of traditional SA restoration, assessing its role in facilitating a carbon-smart agricultural transition.

## Results

### LULC changes

**Fig. 1:**
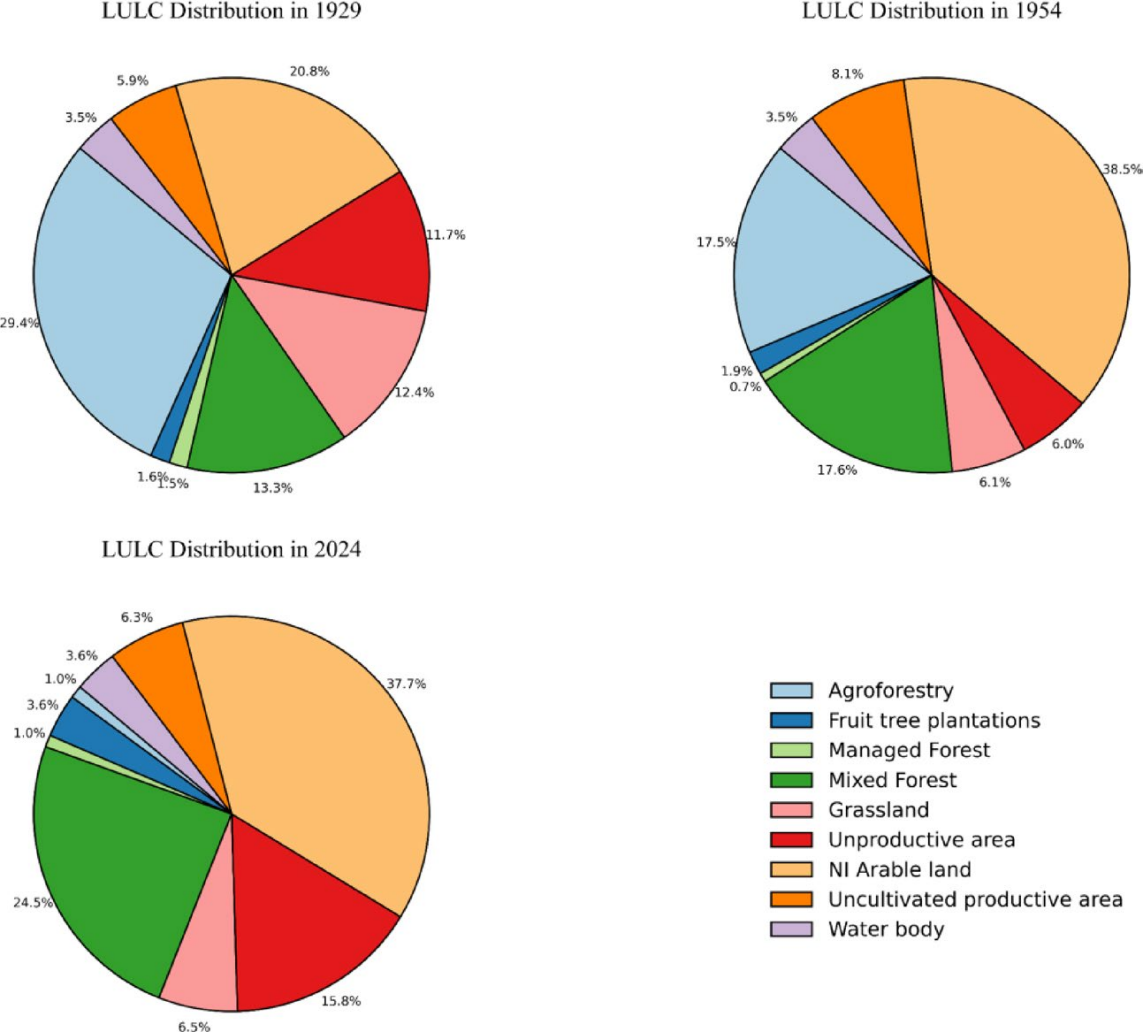
LULC distribution for the years 1929, 1954, and 2024. In this study, “Agroforestry” refers specifically to silvoarable agroforestry systems.

**Fig. 2:**
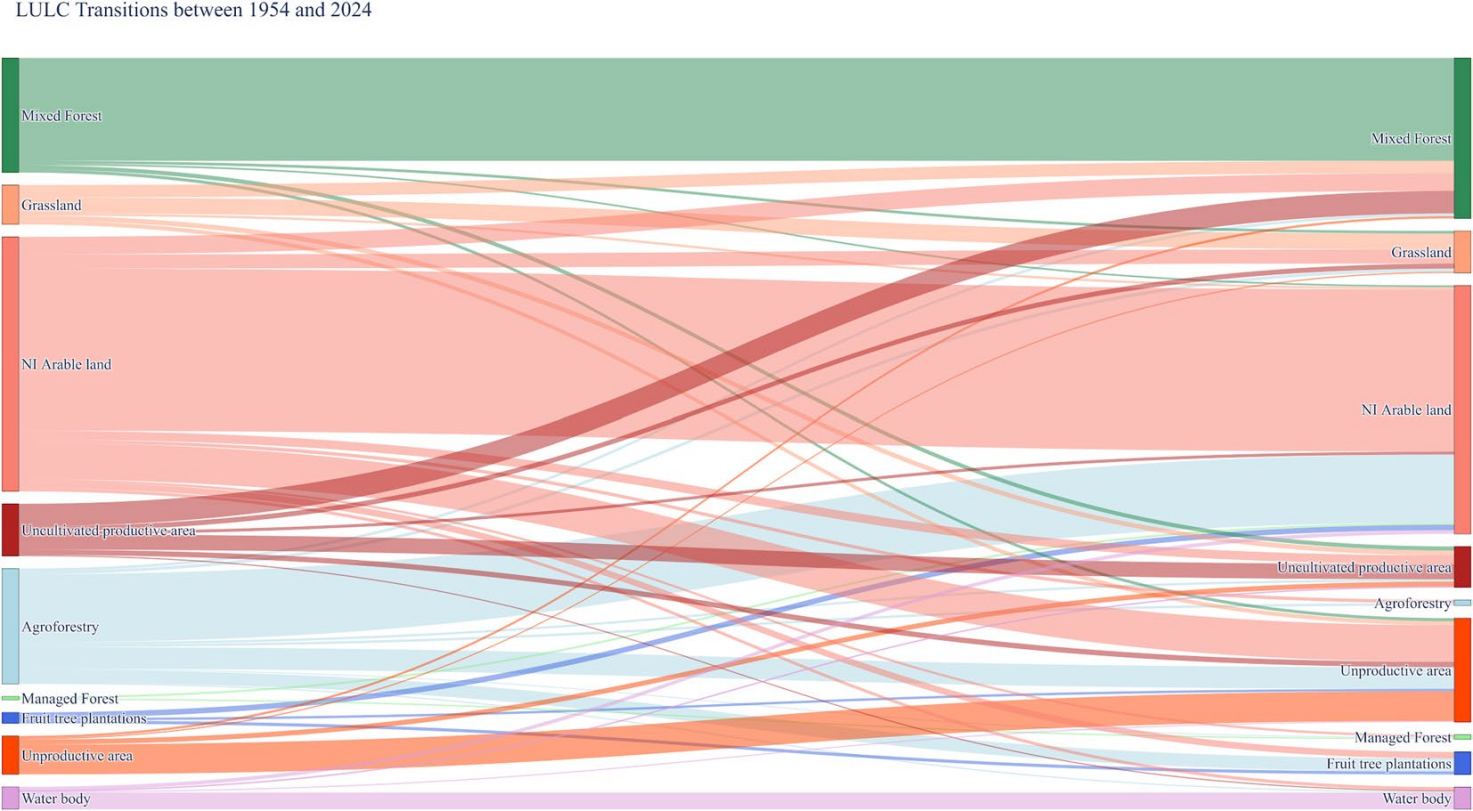
Sankey diagram illustrating LULC transitions between 1954 and 2024. The diagram visualizes changes in LULC types over time, showing shifts such as the transformation of agroforestry areas and the overall landscape dynamics. The thickness of the flows represents the magnitude of transitions between LULC categories. In this study, “Agroforestry” refers specifically to silvoarable agroforestry systems.

Historical records from 1929, 1954, and 2024 (Supplementary Material - B) indicate a near-total disappearance of SA systems, declining from ~ 1.38 Mha to < 50 Kha, while monoculture expanded by 77% over the same period (Table 1; Fig. 1). These dynamics are more evident between 1954 and 2024, as the available data have a precise geospatial reference. At a higher scale, the loss of AF hedgerows is particularly relevant, especially in the Lombardy region (Supplementary Material - A). Furthermore, Fig. 2 precisely tracks LULC transitions from one type to another over the past 70 years.

**Table 1:** Total LULC allocation (ha) for 1929, 1954, and 2024, along with the EU CORINE Land Cover (CLC) nomenclature and corresponding raster values used to generate the 1954 and 2024 geotiffs.

LULC type	LULC allocation 1929 (ha)	LULC allocation 1954 (ha)	LULC allocation 2024 (ha)
Agroforestry	1,382,364	802780.42	47525.03
Fruit tree plantations	75,054	87782.24	163959.79
Managed Forest	69,636	33572.86	45137.78
Mixed Forest	623,929	808314.25	1126240.94
Grassland	582,814	279926.59	296374.59
Unproductive area	550,436	275609.51	725406.93
Non-irrigated arable land	976,838	1767352.22	1730580.29
Uncultivated productive area	276,183	370223.96	289524.22
Water body	162135.1	162135.1	164629.98

In this study, “Agroforestry” refers specifically to silvoarable agroforestry systems.

### CS estimation

To evaluate the effects of these LULC dynamics on carbon sequestration in the region, we developed a statistical model to estimate the associated CS. Due to its near-total disappearance, ISPRA (*Italian National Institute for Environmental Protection and Research*) national GHGs inventory no longer includes AF as a recognized LULC category^56^. However, historical data from *Catasto Agrario 1929*^57^ (hereafter ‘Catasto 1929’) enabled a Monte Carlo (MC) simulation (Supplementary Material - C) to estimate the probable range of AF CS per hectare (t C ha^−1^). The SA system used in this calculation represents the traditional *Coltura Promiscua* documented in historical records. This AF practice consisted of mulberry trees arranged in rows, with grapevines between them, while cereal crops, such as wheat, were cultivated in the spaces between the tree rows.

**Table 2:** Summary of silvoarable agroforestry carbon stock values (t C ha^−1^) estimated using Monte Carlo simulation, historical data, and allometric equations.

Metric	Value (t C ha^−1^)
Min	37.35
Mean	75.38
Median	75.27
Max	122.72
Standard Deviation	13.40
95% CI Lower	50.37
95% CI Upper	101.57

**Fig. 3:**
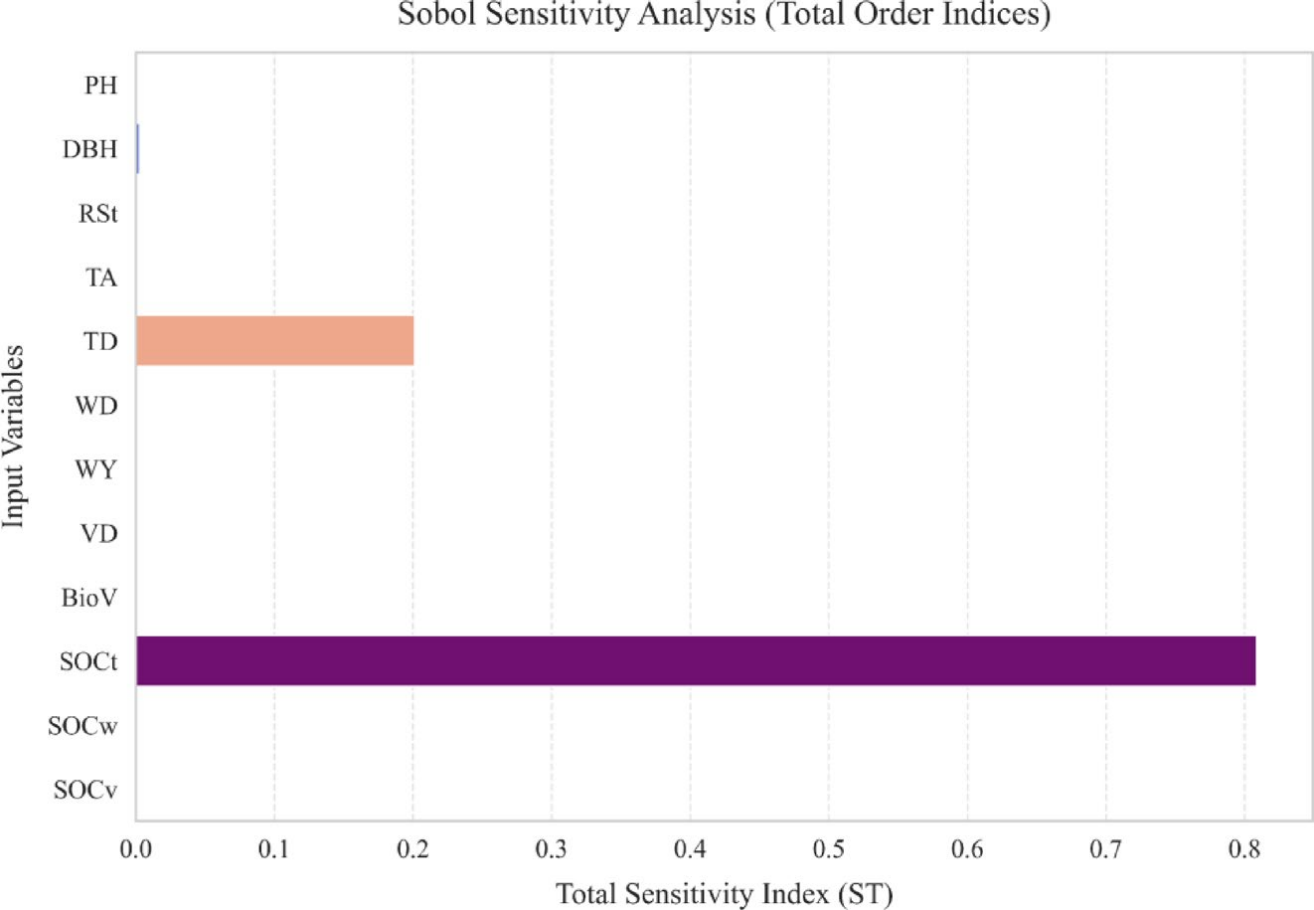
Total-order sensitivity indices (ST) for input variables in the Sobol Sensitivity Analysis. Soil organic carbon of mulberry trees (SOCt) exhibits the highest sensitivity index, indicating its dominant influence on model variability. Mulberry tree density (TD) is the second most influential variable, while mulberry plant height (PH), diameter at breast height (DBH), mulberry wood density (WD), and root-to-shoot ratio (RSt) contribute minimally. Other variables, including wheat yield rate (WY), vine plant density (VD), vine biomass (BioV), soil organic carbon of wheat (SOCw), and soil organic carbon of vines (SOCv), show negligible impact on the model output.

The simulation indicates that AF CS likely ranges from 37.35 to 122.72 t C ha^−1^, with an estimated mean of 75.38 t C ha^−1^, a median of 75.27 t C ha^−1^, and a standard deviation of 13.40 t C ha^−1^ (Table 2). The near-equality of the mean and median in the results indicates that the underlying AF CS distribution is inherently symmetric, strengthening the robustness of the estimates by minimising the impact of extreme values. This is crucial, as a skewed distribution could distort the mean, making it less representative of the central tendency. Moreover, the moderate standard deviation reflects some variability in AF CS, while the 95% confidence interval (50.37–101.57 t C ha^−1^) provides a statistically robust range within which the true values are most likely to fall. To further examine model variability, a Sobol sensitivity analysis^57^ was conducted to quantify the contribution of each input parameter to the overall uncertainty in the results (Fig. 3; Table 3).

**Table 3:** Sobol sensitivity indices for input variables.

Variable	S1	ST
PH	0.00087	0.00161
DBH	0.00203	0.00284
RSt	0	0.00023
TA	0	0
TD	0.18754	0.20132
WD	0.00001	0.00003
WY	0	0
VD	0	0
BioV	0	0
SOCt	0.79487	0.80859
SOCw	0	0
SOCv	0	0

This analysis revealed that the soil organic carbon associated with individual trees (SOCt) is the primary driver of model output variability, exhibiting the highest first-order (S1 ~ 0.79) and total-order (ST ~ 0.80) sensitivity indices. This indicates that nearly 80% of the output variance is attributed to variations in SOCt, highlighting its dominant role in shaping model outcomes. Tree density (TD) emerged as the second most influential variable, with sensitivity indices of S1 ~ 0.18 and ST ~ 0.20, suggesting a moderate impact on model predictions. Conversely biophysical parameters such as plant height (PH), diameter at breast height (DBH) and wood density (WD) contributed minimally to output variance, with sensitivity indices < 0.01. The remaining variables had negligible influence, indicating that they do not substantially affect model variability. These findings underscore the dominant role of SOCt and TD in determining model behaviour, suggesting that CS is largely driven by the number of trees per hectare and the SOC content in correspondence of individual trees.

### Scenario-based simulation

**Fig. 4:**
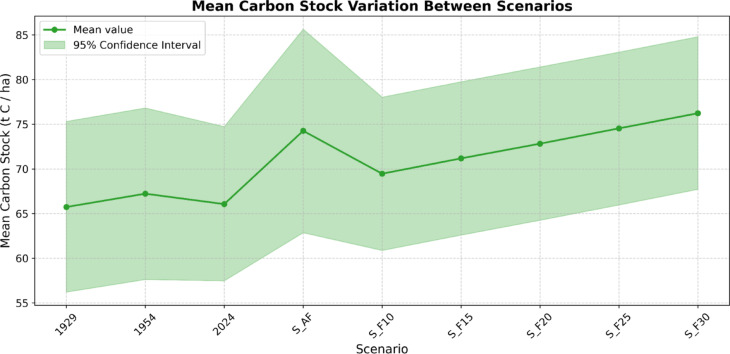
Mean carbon stock (t C ha^−1^) is shown for historical land use periods (1929, 1954, 2024) and future land management scenarios (S_AF, S_F10–S_F30). The solid line represents the mean carbon stock, while the shaded region denotes the 95% confidence interval. The S_AF scenario, simulating full restoration of silvoarable agroforestry, shows the highest projected increase in carbon sequestration. Partial afforestation scenarios (S_F10–S_F30) suggest a gradual increase in carbon storage, with larger afforestation allocations leading to higher sequestration potential.

Finally, the results of the MC scenario-based simulation show how LULC dynamics altered nature-based CS in the area throughout the considered timeframe, as well as the effect of future management strategies (Table 4). Nine scenarios (“Materials and methods” section, Table 5) were simulated by combining the SA-AF CS estimated using historical data with the values provided by ISPRA (“Materials and methods” section, Table 6). As displayed in Fig. 4, CS slightly increased between 1929 and 1954, even though SA-AF had already been replaced by monoculture and urbanisation (Table 1; Figs. 1 and 2). The S_AF scenario, which simulates the complete conversion of 2024 monoculture systems to SA systems, suggests a potential increase in carbon sequestration of approximately 12%. The other management scenarios simulate the partial conversion of monoculture to natural forest (S_F scenarios), with a progressive increase in the land allocated to afforestation (10–30%). To achieve an increase in carbon sequestration like that projected in the full SA-AF reconversion scenario, at least 25% of the current monoculture systems might need to be abandoned to natural afforestation (S_F25).

**Table 4:** Mean carbon stock (t C ha^−1^) and 95% confidence intervals (CI) for different time periods and management scenarios.

Scenario	Mean (t C ha^−1^)	95% CI (t C ha^−1^)	Carbon Seq. (%)
1929	65.69	56.22–75.19	–
1954	67.15	57.59–76.70	2.22
2024	66.05	57.45–74.78	−1.64
S AF	74.19	62.90–85.50	12.31
S_F10	69.48	60.87–78.09	5.18
S_F15	71.14	62.57–79.72	7.70
S_F20	72.84	64.27–81.41	10.28
S_F25	74.54	65.96–83.10	12.85
S_F30	76.22	67.64–84.77	15.39

Historical values are provided for 1929, 1954, and 2024, while future scenarios (S_AF, S_F10, S_F15, S_F20, S_F25, S_F30) represent projected changes under different land management strategies. The percentage of carbon sequestration (Carbon Seq. %) for potential management scenarios is relative to the 2024 baseline, whereas for the historical period, it is calculated as the difference from the preceding period.

## Discussion

In the context of the ongoing climate crisis and global commitments to achieving net-zero emissions by 2050, this study contributes to the broader imperative of developing integrated management strategies and interdisciplinary frameworks to enhance climate resilience through TEK^58^. By leveraging historical LULC data, it reconstructs a real-world, regional-scale SA system that once played a pivotal role in shaping the European rural landscape. In doing so, it bridges the gap between theoretical modelling and practical implementation, offering a concrete, region-specific case study. The integration of historical data with scenario-based modelling provides valuable insights for policymakers and land managers, illustrating how the revival of historic LULC strategies is not limited to landscape heritage conservation, but it serves to promote the transition towards a carbon-smart agriculture.

### Cultural implications of the loss of traditional ecological knowledge

SA-AF in the EU is not merely an innovative practice, but a deeply rooted traditional LU strategy that was systematically dismantled and replaced by mechanised monocultures and urban expansion during the Great Acceleration^29,30^. The LULC transitions reconstructed in this study (Figs. 1 and 2) reflect the socio-economic transformations that unfolded across the agricultural core of Northern Italy throughout the twentieth century CE (Supplementary Material – A). In the lowland areas of the PVP, SA systems such as the *Coltura Promiscua* were increasingly replaced by intensive monoculture and urban development, largely driven by industrialisation and demographic migration from uplands to plains^59^. Meanwhile, upland territories experienced widespread rural depopulation and land abandonment, leading to spontaneous afforestation and the disappearance of traditional land management practices^60,61^.

These dynamics, that have been observed also in other agricultural regions of EU^16^, reconfigured the material structure of local landscape and especially reshaped its epistemic and cultural fabric. TEK, once embedded in everyday practices of land stewardship, has been widely marginalised. In the lowlands, the replacement of site-specific and multifunctional systems with uniform and extractive agricultural models has weakened the socio-ecological feedbacks that historically ensured adaptability and resilience^51,62^. SA-AF systems, integrating ecological function, cultural meaning, and livelihood strategies, once constituted a landscape grammar through which communities expressed identity and belonging. Their disappearance thus represents not only ecological simplification but also cultural rupture.

Not all traditional European AF systems have followed the same trajectory of decline. The SP systems of the Iberian Peninsula (*montado* in Portugal and *dehesa* in Spain) have often been cited as enduring examples of multifunctional, high-nature-value agroecosystems^19,20,63,64^. Their resilience stems from centuries of adaptive management that harmonised ecological processes with rural livelihoods. These landscapes are not only economically productive but also ecologically rich. SP systems appear to have been more resilient than SA systems in withstanding the socio-economic and environmental transformations associated with the Great Acceleration, which led to the widespread replacement of traditional SA systems with mechanised monocultures across much of the EU. However, recent research indicates that even this relative resilience is increasingly under growing pressure. According to Pinto-Correia et al.^65^ the economic importance of the *montado* in Portugal is in decline, despite its continued role as a multifunctional system. Long-standing adaptive management practices that once integrated ecological complexity with rural livelihoods are now being undermined by shifting governance regimes and conflicting LU discourses^65^. Although these landscapes still provide significant ecological and cultural value, SP systems are now facing many of the same pressures that contributed to the disappearance of SA systems. This situation exemplifies the broader tensions involved in efforts to preserve TEK within contemporary socio-political and market-oriented frameworks, highlighting the need for proactive strategies to safeguard this intangible cultural heritage.

In Italy, as elsewhere, traditional agricultural landscapes are now recognised as part of the national cultural heritage. The National Register of Historical Rural Landscapes, established by the Ministry of Agriculture^66^ identifies 123 areas characterised by long histories of LU continuity, high biocultural diversity, and complex landscape mosaics. These areas, often shaped by centuries of traditional and sustainable AF practices, represent multifunctional spaces where ecological processes, local economies, and cultural identity co-evolved^67^. Yet, more than 10 million hectares of agricultural land have been abandoned in the past century, and spontaneous reforestation now covers over one-third of the national territory. The fresh expansion of forests is not the regeneration of the pristine local land cover and represents ecosystems altered by humans^68^ including the introduction of invasive alien species^69^. The major consequences are the degeneration of ecosystem services and the drastic reduction of landscape heterogeneity and its historical legibility. This spatial homogenisation obscures the “story of place,” severing the connection between rural communities and their environmental heritage^70^.

The implications of this loss are reflected in the weakening of cultural resilience. The identity of traditional rural landscapes lies in the coherence of their composing elements; once these are fragmented or removed, the ability to interpret and value the landscape erodes. The collapse of TEK systems thus contributes to a broader crisis in rural landscape heritage legibility, where the symbolic, aesthetic, and historical dimensions of place are rendered invisible.

### The impact of historic LULC changes on carbon sequestration

By tracking long-term LULC dynamics, this study highlights how these shifts have directly impacted the region’s nature-based carbon sequestration, altering its capacity to store carbon. The MC simulation employed in this study offers a robust estimation of the carbon storage potential of the historic *Coltura Promiscua* system by integrating historical rural data, allometric equations, and statistical validations (Supplementary Material - C). The resulting estimates suggest that well-preserved SA systems could have stored considerable amounts of carbon, with an upper bound of 122.72 t C ha^−1^, while the lower bound of 37.35 t C ha^−1^ reflects the influence of more degraded or sparsely vegetated SA remnants (Table 2). By demonstrating both statistical robustness and an approximately symmetric distribution, these results provide a reliable basis for assessing the historical carbon storage potential of the now-disappeared *Coltura Promiscua* SA system. Furthermore, the overlap between the confidence interval and literature values reinforces the robustness of these estimates. The 95% confidence interval (50.37–101.57 t C ha^−1^, Table 2) aligns well with values reported in empirical studies on European SA-AF systems^71,72^ indicating that the estimated range from the MC simulation is consistent with observed CS variability in managed SA landscapes. Furthermore, the scenario-based approach yields valuable insights into the effects of LULC changes on CS over the past century. Particularly noteworthy is the observation that, despite the widespread abandonment of SA-AF between 1929 and 1954, carbon sequestration capacity exhibited a slight increase (Table 4; Fig. 4). This trend likely reflects the compensatory effect of natural afforestation, largely driven by the abandonment of agriculture and depopulation in uplands, a phenomenon that may have continued to offset the loss of SA-AF until the present day. Indeed, over the past 70 years, only a ~ 1.6% reduction in LULC carbon sequestration has been recorded (Table 4; Fig. 4). Although historical variations in carbon sequestration suggest that the complete loss of SA-AF has had minimal impact on CS at a regional scale, the management scenarios reveal a different trend. A scenario simulating the total recasting of historic SA-AF (S_AF) indicates that regional carbon sequestration capacity could increase by up to ~ 12%. Scenarios incorporating partial afforestation of rural areas (S_F10 to S_F30) provide further valuable insights for potential future carbon-smart agriculture policies. Notably, allocating only a quarter of the total rural area to afforestation (S_F25) appears to achieve the same carbon sequestration benefits as a scenario simulating the full conversion of contemporary monoculture to SA-AF (Table 4; Fig. 4). In terms of enhancing nature-based CS, both solutions appear to yield comparable benefits. However, between the complete recasting of historical SA systems and the partial afforestation of current farmland, there exist significant environmental, ecological, and economic implications that must be carefully addressed.

### Challenges and opportunities for recasting historic silvoarable agroforestry

A key barrier to the widespread adoption of SA-AF is the limited understanding of optimal tree-crop combinations, which often constrains large-scale implementation. By reconstructing the traditional *Coltura Promiscua*, this study provides essential insights into species selection, planting densities, and LU configurations that could inform the design of modern SA systems tailored to regional needs. A second major concern among farmers regarding the adoption of AF systems relates to agricultural productivity. Although studies in tropical regions suggest that yield trade-offs exist (system yields are often higher in organic SA-AF, yet individual crop yields tend to be lower than in monocultures^73^), the dynamics in temperate regions appear to differ. In temperate SA systems, overall productivity generally surpasses that of monocropping due to the complementary use of resources between trees and crops. Trees contribute to enhanced nutrient cycling, improved water retention, and greater microclimate stability, creating more favourable crop growth conditions while reducing input requirements^74^. Historical data from 1929^57^ supports this perspective, indicating that SA-AF wheat yield rates were comparable to those of monoculture systems (mean wheat yield in 1929: monoculture, 1.449 t ha^−1^, SA-AF, 1.450 t ha^−1^). These insights challenge the conventional view that SA-AF is inherently less productive than monoculture, reinforcing its potential role in land-sharing strategies that integrate conservation with agricultural production^75^. While afforestation provides a long-term carbon storage mechanism, SA-AF offers an immediate and adaptive solution by integrating trees within productive agricultural landscapes. Unlike afforestation, which necessitates the conversion of farmland into forests, SA-AF enables simultaneous carbon sequestration and agricultural production, expanding natural carbon sinks while sustaining rural livelihoods.

A further key distinction between afforestation and SA-AF lies in tree growth dynamics. In forestry plantations, tree height increases gradually over approximately 30–50 years^76^ after which growth slows due to canopy closure and resource limitations. Fast-growing species, such as poplar, alder, typically reach peak growth within 30–40 years, whereas slower-growing species, such as oak and beech, may require 60–80 years to mature^77^. Long-term growth in forestry systems is generally slower due to intense competition for light and nutrients in densely planted stands. In contrast, SA systems (especially those incorporating mulberry^78–80^), although initially characterised by slower tree growth, often surpass forestry plantations in both height and diameter over time, with significant differences observed as early as five years after planting. This accelerated development is attributed to improved soil quality, enhanced nutrient cycling, and diversified LU^81^. Consequently, SA systems can reach their carbon sequestration plateau earlier than conventional forestry systems, typically within 20–30 years^77^.

The variation in tree growth rates and productivity should serve as strong incentives for prioritising SA-AF over afforestation as a multipurpose, carbon-smart agricultural strategy. However, the choice between these approaches is not straightforward. There is a recognised need for flexibility in carbon offset initiatives, particularly in selecting between SA-AF and afforestation models and adapting approaches to regional contexts. A regionally adaptive strategy is essential, as SA-AF and afforestation should not be viewed as mutually exclusive, but rather as complementary approaches tailored to specific landscapes and communities^45^.

In temperate regions, a mixed strategy combining land-sparing and land-sharing is often necessary due to the heterogeneous landscapes, agricultural history, and existing biodiversity patterns. Neither a purely land-sparing nor a purely land-sharing approach is optimal. Instead, an integrated strategy that accounts for species requirements, ecosystem services, and economic factors is more effective in achieving sustainable land management^82^. Furthermore, understanding the supply costs of carbon sequestration is key to designing market-based mechanisms that encourage SA-AF. The economic feasibility of SA practices, particularly those that minimise LU conversion, highlights their suitability for voluntary carbon markets, which may better serve small-scale farmers in rural areas. Recent initiatives, such as the ACORN Platform or the Dream Fund Program, have shown that targeted support can improve smallholder access to carbon finance through simplified certification and robust monitoring systems^12^. The new EU certification framework (Regulation 2024/3012) further reinforces the role of carbon farming in climate policy, providing a standardised but flexible system for recognizing soil-based removals, while also promoting additionality and biodiversity co-benefits^13^. However, challenges around permanence and credibility persist, especially within voluntary markets that have so far overrelied on emission avoidance rather than verified removals^11^. Additionally, SA practices, not requiring full land conversion, provide cost-effective solutions with lower opportunity costs. For large-scale implementation, policies should prioritise financing strategies that encourage participation and long-term sustainability. Without sufficient financial support, landowners may be unfairly burdened with the costs of climate mitigation^83^. Continued research is necessary to refine cost estimates and improve sequestration methodologies, ensuring that SA-AF becomes a cornerstone of climate change mitigation while delivering both environmental and economic benefits.

### Future directions

Although our scenario analysis focuses on landscape-scale trends, it is important to recognize that SOC associated with individual trees (SOCt) plays a key role in driving the CS values observed in SA-AF systems. This underscores the need for ground-truthing SOCt through field-based measurements. Since SOC accumulation is highly variable at the local scale - depending on soil type, climate, tree species, LU history, and management - future research should prioritize site-specific SOC sampling and regionally calibrated models to refine CS assessments and better inform nature-based policy decisions.

The economic impact of full SA-AF conversion (scenario S_AF) remains uncertain, particularly in the short to mid-term. One key challenge is labour intensity: SA-AF could require more labour input, which may hinder adoption unless fair compensation or premium pricing mechanisms are in place^73^. Although SA-AF demands higher initial investment and labour, it typically yields greater long-term profitability and environmental benefits. The increased labour cost stems from tree maintenance, diversified activities, and reduced mechanisation. However, over time, economic returns from timber, livestock, and crops might offset these costs^74^. Future studies should further explore the short- and mid- term economic sustainability of SA-AF, particularly in balancing labour demands with financial incentives and market viability.

While SA-AF offers significant carbon sequestration potential, its widespread adoption hinges on financial viability. Future studies should assess productivity shifts, upfront investment costs, and revenue diversification under SA systems. Additionally, evaluating carbon sequestration rates per hectare will be essential for informing incentive structures and carbon credit schemes that could drive broader SA-AF implementation. The recent EU Regulation 2024/3012 provides a harmonised certification framework that can support soil-based carbon removals and reward additional efforts in carbon farming while safeguarding biodiversity^13^. However the current structure of voluntary markets often emphasises emission avoidance over verified removals, limiting their effectiveness unless transparency and standardisation improve^11^. Beyond carbon sequestration, SA-AF plays a pivotal role in restoring degraded agricultural landscapes by enhancing biodiversity, improving ecological connectivity, and strengthening landscape resilience^75,84^. Further research should also assess how different SA-AF configurations influence biodiversity at various scales and identify strategies to maximise ecological benefits while maintaining agricultural productivity.

Finally, our findings can be interpreted on a broader scale, extending beyond EU borders, to underscore the enduring value of SA systems. These practices remain widespread in many low-income countries, where they are increasingly under threat from the global expansion of industrialised agriculture. A key insight is that in such regions, a shift towards intensive agricultural methods is not necessarily positive, as these often lead to soil degradation and a long-term decline in productivity. By contrast, the preservation and integration of TEK, such as SA practices, could support comparable yields, while simultaneously enhancing ecosystem services, safeguarding soil resources, and promoting significant CS.

## Materials and methods

The methodological protocol employed has been designed using free and open-source software (FOSS) wherever possible, promoting reusability, reproducibility, and adaptability.

**Fig. 5:**
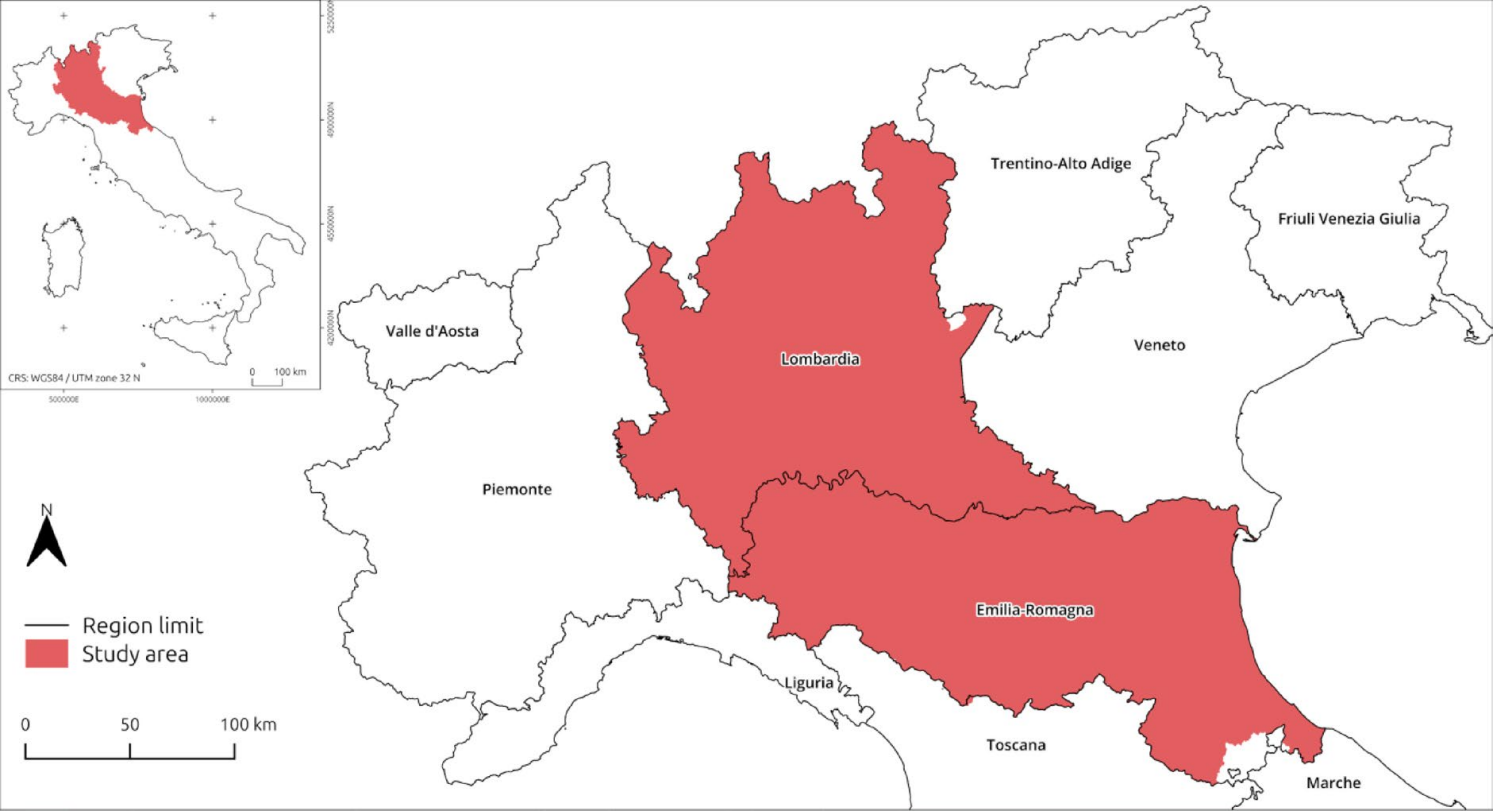
Location of the study area (Image generated with the software QGIS 3.40 LTR (https://www.qgis.org/en/site/index.html)).

Data were collected from the two administrative regions of Lombardy and Emilia-Romagna (Fig. 5). Several reasons guided this choice. First, data consistency: for both regions, it was possible to retrieve geospatial information about LULC from the time frame considered (1929–2024), whereas for others, this was not always feasible. Secondly, historically, the traditional AF of *Coltura Promiscua* was widely practised especially in Lombardy and Emilia-Romagna until twentieth century CE. Thirdly, these two regions together account for approximately 70% of the total area of the PVP^85^ making the data collected here a sufficiently representative sample of historical LULC changes across the PVP.

The entire workflow has been developed in Python. The script is divided into five steps to keep each phase of the workflow separate. The entire procedure can be executed using the *pipeline.py* script, which iteratively runs all five steps of the process. Values, parameters, and directory paths used in the workflow are defined in the *config.py* file to ensure consistency and facilitate code updates. The complete Python procedure is fully available on Zenodo at: 10.5281/zenodo.13929576.

The first two steps (*step_1.py*, *step_2.py*) of the Python protocol involve dataset development. Three distinct periods were considered in the analysis—the 1929, 1954, and 2024—covering almost 100 years of LULC changes in the study area. Information on the rural landscape of the study area in 1929 is based on the Catasto 1929, a survey carried out by the Italian National Institute of Statistics (*Istituto Nazionale di Statistica* – ISTAT) between 1928 and 1930, covering the entire Italian territory^86^. This survey produced a dedicated volume for each Italian region, providing detailed descriptive data on the rural landscape of the time. It serves as a comprehensive inventory, offering information on agricultural and forested areas, including the mean number of trees per hectare employed in AF systems, LU by individual crops, crop species, and average crop yields per hectare^87^. The 1954 LULC data was obtained from historical aerial imagery collected during the *Volo Base Gruppo Aeronautico Italiano*, Italy’s first nationwide stereoscopic and planimetric survey, coordinated by the *Istituto Geografico Militare*. The 2024 LULC dataset is based on aerial imagery provided by AGEA (*Agenzia per le Erogazioni in Agricoltura*). The vector datasets for both 1954 and 2024 were retrieved from their respective regional geodatabases^88,89^.

The *step_3.py* script uses the 1929’s municipality vector layers to extract LULC data from the 1954 and 2024 raster files. It then returns the total area allocated to each category for the three periods (1929, 1954, and 2024). The total area (measured in ha) allocated to each LULC category across the three periods is summarised in Table 1. Further details on dataset development are provided in Supplementary Material A.

The *step_4.py* script performs MC simulations and sensitivity analysis to estimate SA-AF CS based on biophysical parameters, using geospatial data filtering and statistical analysis. According to the InVEST Model approach^90^ the CS value of a LULC type is derived from the sum of its four carbon pools: above-ground carbon (AGC), below-ground carbon (BGC), dead matter (DM), and soil organic carbon (SOC). In this study, values were obtained from existing literature and allometric equations. The SA system was modelled based on the *Coltura Promiscua* main traditional setting comprising mulberry trees, wheat, and grapevines. To improve data reliability, outliers were filtered using trimmed mean and median tolerance methods^91^ depending on the skewness of the dataset. CS values were estimated using MC simulations, with the optimal number of simulations determined via the Central Limit Theorem^92^ to ensure statistical robustness (details provided in Supplementary Material B).

Furthermore a Sobol sensitivity analysis^93^ was conducted to quantify the contribution of each key variable to the variance of SA-AF CS. Sobol analysis is a global sensitivity analysis method based on variance decomposition, widely used in uncertainty quantification and model evaluation. This approach enables a comprehensive assessment of how input variables influence output variance, accounting for both independent effects and complex interactions. It is particularly advantageous for nonlinear and non-additive systems, making it well-suited for environmental modelling and ecological research^57^. Sobol sensitivity analysis enables the calculation of three indices. First-order indices (S1) measure the direct contribution of each variable to the variance of SA-AF CS, indicating its independent effect. A high S1 value suggests that the variable alone significantly impacts the output. Total-order indices (ST) represent the overall influence of each variable, accounting for both its direct effect and interactions with other variables. A high ST value indicates that a variable’s impact is amplified when interactions are considered. Second-order indices (S2) capture pairwise interaction effects between variables, revealing synergistic or compensatory relationships^93^. These indices help assess how two variables together influence SA-AF CS variance beyond their individual contributions.

The final step of the Python script (*step_5.py*) analyzes LULC changes and their impact on CS by simulating different scenarios. Nine scenarios were employed: three temporal scenarios representing LULC in 1929, 1954, and 2024, respectively, and six management scenarios. The management scenarios simulate: (i) the total conversion of *Non-Irrigated Arable Land* to *Silvoarable Agroforestry* (S_AF), and (ii) the partial conversion of *Non-Irrigated Arable Land* to afforestation, ranging from 10% (S_F10) to 30% (S_F30) (Table 5).

**Table 5:** Description of the temporal and management scenarios used in the analysis.

Type	Name	Description
Temporal Scenario	1929	Historical land use and land cover representing the landscape configuration in 1929
	1954	Historical land use and land cover representing the landscape configuration in 1954
	2024	Present-day land use and land cover configuration
Management Scenario	S_AF	Total conversion of *Non irrigated arable land* to *Agroforestry* (i.e. 100% *Agroforestry*, 0% *Non irrigated arable land*)
	S_F10	Afforestation of the 10% of 2024 *Non irrigated arable land* (i.e. − 10% *Non irrigated arable land* → + 10% *Mixed Forest*)
	S_F15	Afforestation of the 15% of 2024 *Non irrigated arable land* (i.e. − 15% *Non irrigated arable land* → + 15% *Mixed Forest*)
	S_F20	Afforestation of the 20% of 2024 *Non irrigated arable land* (i.e. − 20% *Non irrigated arable land* → + 20% *Mixed Forest*)
	S_F25	Afforestation of the 25% of 2024 *Non irrigated arable land* (i.e. − 25% *Non irrigated arable land* → + 25% *Mixed Forest*)
	S_F30	Afforestation of the 30% of 2024 *Non irrigated arable land* (i.e. − 30% *Non irrigated arable land* → + 30% *Mixed Forest*)

The Temporal scenarios represent historical and current LULC distributions.

The management scenarios explore different levels of afforestation, with a complete conversion (*S_AF*) to *Agroforestry* and partial conversions (*S_F10–S_F30*) of *Non-Irrigated arable land to Mixed Forest*.

To estimate the carbon sequestration potential of these scenarios, the script applies MC simulation, which generates random values within defined ranges for each LULC type. These simulations enable the estimation of total and mean CS values under different LULC scenarios, incorporating uncertainty in CS values. The LULC type CS ranges used in the MC simulations were obtained from the *Italian National Institute for Environmental Protection and Research* (ISPRA) – *Greenhouse Gas Inventory Report 2024*^56^, except for SA-AF, for which the 95% confidence interval values estimated in *step_4.py* were used. The ISPRA carbon pool LULC type ranges were defined using the minimum and maximum values for the Lombardy and Emilia-Romagna regions. Since detailed information about rural land management in 1929 and 1954 is uncertain, to account for these uncertainties in the simulations across different temporal scenarios, all management strategies and plant species described in the ISPRA report were considered^56^. Ranges for *Unproductive area* and *Uncultivated productive area* were estimated from a similar case study that adopted the InVEST Model approach^94^ while the contribution of *Water Body* was considered as zero (Table 6).

**Table 6:** Carbon stock ranges for LULC types used to estimate carbon sequestration between different scenarios.

LULC type	Carbon stock ranges (t C ha^−1^)	Source
Silvoarable-Agroforestry	50.37–101.57	*MC simulation*
Fruit Tree Plantations	38.3–86.59	D. Romano et al. 2024
Managed Forest	88.32–99.15	D. Romano et al. 2024
Mixed Forest	126.88–161.17	D. Romano et al. 2024
Grassland	61.75–89.81	D. Romano et al. 2024
Non-irrigated Arable_land	36.64–71.88	D. Romano et al. 2024
Unproductive area	1–9	X. Li et al. 2022
Uncultivated productive area	1–20	X. Li et al. 2022
Water body	0	–

Finally, carbon sequestration was estimated as the percentage difference between scenarios. For the temporal scenarios, carbon sequestration represents the percentage difference between two consecutive periods (1929, 1954, 2024). For the management scenarios (S_AF, S_F10, S_F15, S_F20, S_F25, S_F30), carbon sequestration was calculated from the 2024 scenario to estimate the potential future carbon benefits that land management choices could have on the regional nature-based CS.

## Supplementary Information

Below is the link to the electronic supplementary material.

Supplementary Material 1

## Data Availability

The data and the Python script code used in this study are fully available on Zenodo at the project RhECAST repository: https://doi.org/10.5281/zenodo.13929576.
