# Supplementary material for: Data-driven scenario analysis supports the revival of historic silvoarable systems for carbon smart rural landscapes
Source: Sci Rep. 2025 Oct 7;15:34963. doi: 10.1038/s41598-025-18950-7 (PMC12504591; doi:10.1038/s41598-025-18950-7)
Supplement: Supplementary file 1 — Supplementary Material 1 [file 41598_2025_18950_MOESM1_ESM.pdf]

Supplementary Material

A- Study Area

This study focuses on the Po-Venetian Plain (PVP, Northern Italy), one of the most polluted and densely populated regions in Europe [1]. Covering approximately 46,000 km^2, the PVP lies primarily within the Po River basin, bordered by the Alps, the Apennines, and the Adriatic Sea [2,3] (Fig. S1).

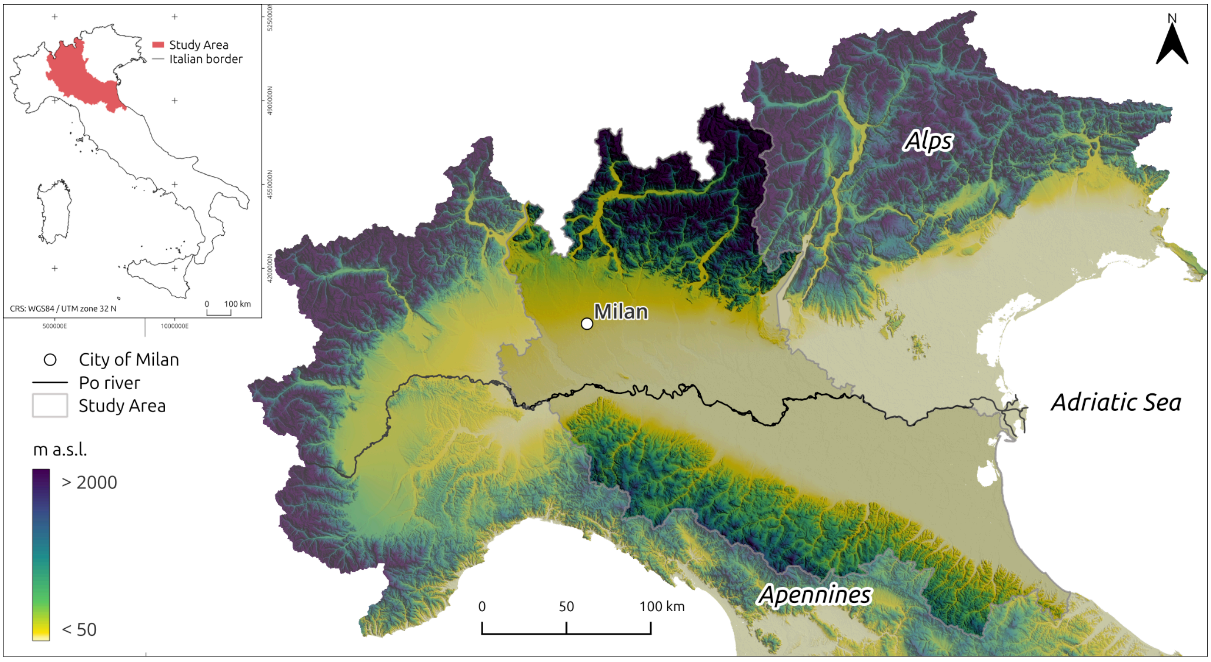

Fig.S1 - Topographical setting of the PVP and location of the study area (Image generated with the software QGIS 3.40 LTR (https://www.qgis.org/en/site/index.html)).

The region's mountainous topography restricts air circulation, contributing to Milan being one of the world's major hotspots for PM_{10} and atmospheric greenhouse gases (GHGs) [4]. The PVP accounts for nearly 55% of Italy's total agricultural GHGs emissions, with CO_2 contributing approximately 20%. Over the past 15 years, GHGs emissions have declined slightly across all economic sectors in Italy. However, the agricultural sector has shown the smallest reductions, with emissions projected to remain stable until 2030 [5]. The environmental history of the PVP has been shaped by complex settlement patterns and land managing strategies [6], including early evidence for crop rotation and water management at least since the Late Holocene (~3500 BCE)[7,8]. Between the 15th and 19th centuries CE, the primary rural strategy was a traditional agroforestry (AF) system, known as *Coltura Promiscua*, which consisted of long arable strips separated by rows of trees supporting trained vines [9]. This traditional AF system was extensively adopted across the PVP and the northern Apennines, where it was known by various regional names reflecting distinct local variants and technical practice[10,11].

The earliest known agronomic reference to *Coltura Promiscua* dates to 1495 CE, when Piero de' Crescenzi described mixed cultivation practices in his treatise *De agricultura*. Subsequent historical records continued to document this traditional system of intercropping. In 1580, Agostino Gallo explored similar agricultural methods in *Le vinti giornate dell'agricoltura, et de' piaceri della villa*. By 1622, Bernardo Davanzati provided further insights into rural practices involving the cultivation of vines and trees in his *Coltivazione toscana delle viti e d'alcuni alberi*. Later, in 1766, J.J. Le Français de Lalande, during his travels through Italy, reported on the widespread use of *Coltura Promiscua* in

his *Voyage d'un François en Italie, fait dans les années 1765–1766*. Finally, in 1801, Simonde de Sismondi offered a detailed account of the system in his work *Tableau de l'agriculture Toscane*, highlighting its enduring role in local agronomic traditions. A comprehensive review of these historical records is presented in the seminal mid-20th-century work of Emilio Sereni [12], which offers a foundational introduction to the origins and evolution of *Coltura Promiscua*, along with detailed data on the regional distribution of traditional AF at the time.
Traditional AF system was particularly prevalent in the administrative regions of Lombardy and Emilia-Romagna, where it was recorded in historical documents as *piantata padana* and *piantata emiliana*, respectively (Fig. S2).

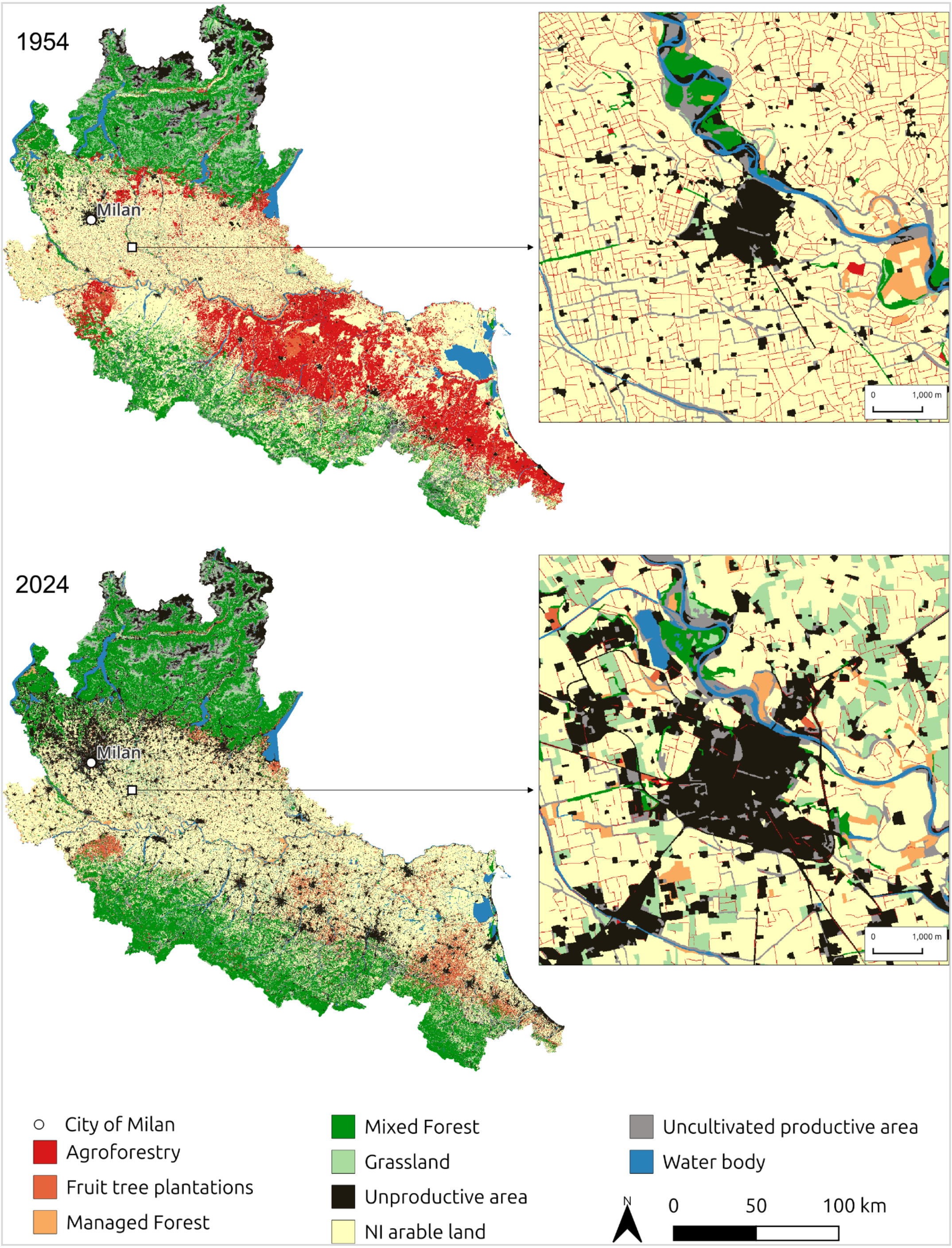

Fig. S2 - Spatial distribution of land use and land cover LULC types in 1954 (top) and 2024 (bottom). The enlarged area highlights the significant disappearance of hedgerows over the past 70 years (Image generated with the software QGIS 3.40 LTR (https://www.qgis.org/en/site/index.html)).

According to historical sources, the extent of AF in the PVP was approximately 1,281,000 hectares in the mid-19th century, increasing to 1,932,000 hectares by 1911, when it reached its peak. In the decades that followed, it progressively declined to 1,480,000 hectares in 1929, 1,344,000 hectares in 1949, and 1,196,000 hectares by 1957 [12]. This downward trend marked the beginning of a steady decline, and following its peak in the 19th century CE, the traditional AF system was progressively abandoned throughout the 20th century [13,14]. Since the 1950s, this process has accelerated, as the PVP landscape underwent an unprecedented transformation due to rapid urbanisation and agricultural mechanisation, leading to the widespread replacement of traditional AF with monoculture[15]. This land-use shift occurred within the broader context of the post-World War II *Great Acceleration*, a period of rapid, human-induced global environmental change [16]. In particular, the study region was further affected by agrarian reforms of the 1940s and 1950s [17], which reshaped Italy's agricultural landscape—especially in the lowlands—by promoting rural intensification and modernising farming structures. Meanwhile, in the uplands, rural depopulation led to natural forest regeneration [18,19] (Fig. S2). Together, these environmental, socio-economic, and historical dynamics position the PVP as an ideal case study for assessing the impact of AF abandonment on nature-based carbon stocks, as well as for simulating the potential benefits of AF restoration in the region.

B - Dataset Development

The dataset covers land use and land cover (LULC) changes in the Lombardy and Emilia Romagna regions in the years 1929, 1954 and 2024. The data retrieved from the Catasto 1929 were digitised through a semi-automatic process and subsequently imported into GIS to develop geospatial datasets in vector format. The dataset was created following a rigorous protocol to ensure consistency throughout its compilation. The first step involved extracting information from the original rural registry. Digital scans of the Catasto 1929 volumes were retrieved from the ISTAT official repository [20]. The data were extracted semi-automatically using the Optical Character Recognition (OCR) software FineReader PDF 16 [21]. After creating a training dataset of over 5,000 characters, the software was used to extract tabular information and convert it into a spreadsheet file format (Fig. S3).

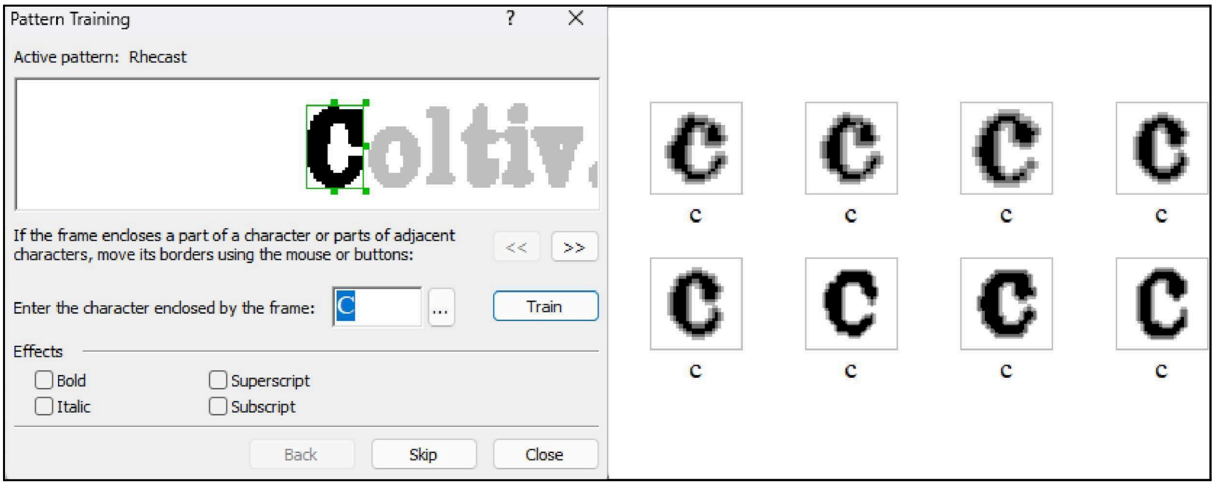

Fig. S3. On the left: the training tool in FineReader PDF 16 used to create the training dataset for the automatic digitization of the Catasto Agrario 1929. On the right: an example from the training dataset, showing eight elements that represent the letter 'C'.

The second step focused on creating an empty geospatial dataset representing both municipalities and provinces of the study area as they existed in 1929. Over the past 95 years, Italy's territorial administrative boundaries have undergone several changes, including splits, mergers, and renaming. Therefore, it was essential to identify areas that no longer aligned with the administrative divisions of 1929. Each volume of the Catasto 1929 included a map of the administrative area from which the data were collected. These maps were georeferenced using the software QGIS [22] and a 'backdating approach' [23,24] to identify discrepancies between the 1929 and present-day administrative boundaries and place names (Fig. S4). To ensure accurate georectification of the 1929 maps, at least three ground control points were identified [25]. The Thin Plate Spline (TPS) and cubic resampling

methods were then applied [26]. Additionally, vector layers representing present-day Italian municipalities and provinces were obtained from the National Geodatabase [27] and modified to reflect the administrative divisions of 1929. The final vector layers were exported in Geopackage (GPKG) file format. The GPKG format was chosen for its standards-based, platform-independent, and compact structure, facilitating the efficient transfer and reuse of geospatial data [28].

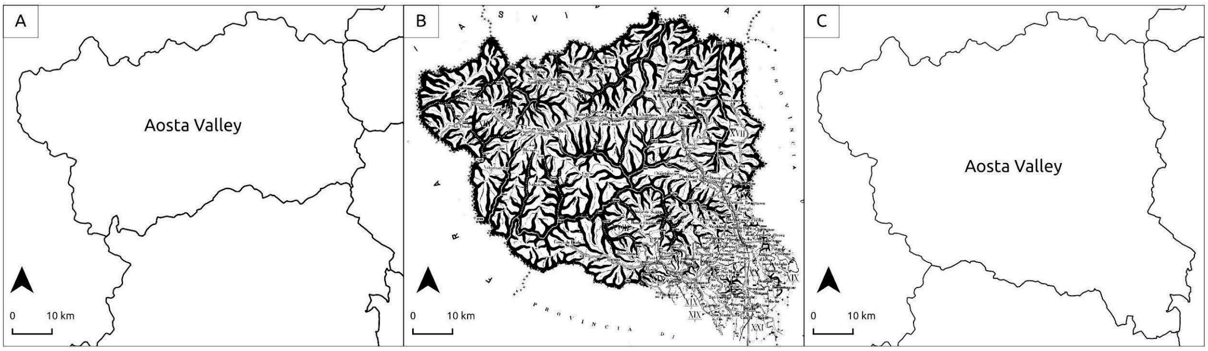

Fig. S4. Example of the differences in provincial administrative boundaries between the 1920s and the present day. A) The current extent of Aosta Valley province; B) The Catasto Agrario 1929 map with Aosta Valley province georeferenced in QGIS; C) The vector layer of Aosta Valley province reflecting the boundaries in the 1920s (Image generated with the software QGIS 3.40 LTR (https://www.qgis.org/en/site/index.html)).

The third step involved transferring the information extracted through OCR from the 1929 rural registry into the empty GPKG files. To achieve this, a Python script was developed to automatically extract data from the OCR-generated spreadsheets, create table fields, and organise the information for each municipality and province (*step_1.py*). This script ensured consistency in database development and was built using two widely used Python libraries for data manipulation and geospatial data management: `Pandas` [29] and `GeoPandas` [30]. The final outputs consist of updated GPKG files detailing the rural landscape of the study area in 1929 at both the municipal and provincial levels. The last step involved dataset validation. To prevent topological errors, the QGIS `Topology Checker` plugin was used to identify potential spatial inconsistencies such as overlaps, slivers, or duplicates. These issues were then resolved using the GRASS module `v.clean` [31].

The EU CORINE Land Cover (CLC) nomenclature [32] was used to classify the LULC types recorded in the Catasto 1929. The LULC categories included in this dataset are: agroforestry (CORINE class 2.1.1), fruit tree plantations (CORINE class 2.2.2), pasture (CORINE class 2.3.1), non-irrigated arable land (CORINE class 2.4.4), grassland (CORINE class 3.2.1), managed forest (CORINE class 3.1.1), mixed forest (CORINE class 3.1.3), and water body (CORINE class 5.1.2). Additionally, two supplementary classes were introduced: *uncultivated productive area* and *unproductive area*. The former corresponds to areas recorded in the Catasto 1929 as *Incolti produttivi*, referring to land potentially suitable for agriculture but uncultivated at the time of the survey. The latter includes all remaining areas within each municipality/province that were not classified in the Catasto 1929, encompassing geologically non-productive areas (e.g., rock outcrops), water bodies (e.g., lakes and rivers), and infrastructure (e.g., buildings, roads, and railways). The selection of these categories ensures better alignment with the structure of the Catasto 1929. For instance, in the Catasto 1929, woodland coverage was generally recorded without specifying dominant forest species (e.g., broadleaf or coniferous), leading to its classification as *mixed forest* in this dataset. Conversely, chestnut groves, when explicitly mentioned in the Catasto 1929, were recorded separately as *managed forest*.

Information on LULC for 1954 and 2024 is available in vector file format within regional databases [33,34]. Both regions periodically update their respective LULC datasets based on aerial observations. The original vector files for both 1954 and 2024 were retrieved from the regional databases and subsequently processed using Python *(step_2.py*). To ensure comparability with the 1929 dataset, the LULC categories from the regional geodatabase were grouped according to the CLC nomenclature. Also, since the information from 1929 lacks spatial resolution and only represents the allocation of LULC types within administrative boundaries, it was necessary to manipulate the 1954 and 2024 data to extrapolate LULC allocations using the same vector layers representing 1929

municipalities/provincies. This enabled a comparison of LULC allocation across the three different periods. First, the *step_2.py* script converts LULC vector data from GPKG format into raster format. In addition, the Lombardy regional geoportal provides vector file data on the coverage of hedgerows for both 1954 and 2024. Hedgerows are a distinctive component of AF systems that enhance nature-based carbon storage in rural areas [35–37]. In Lombardy, they were a crucial element of the local AF system known as *piantata padana* [12], making it necessary to include them in the development of the LULC raster for the research area in 1954 and 2024. The results of *step_2.py* are two raster GeoTIFF files representing the LULC of 1954 and 2024 (Fig. S2).

The third phase of the Python procedure (*step_3.py*) involves extracting the LULC allocation in hectares (ha) for the three different periods. To ensure comparability across the three datasets, it was necessary to estimate the extent of the CLC category "water body" (CORINE class 5.1.2) for the 1929 dataset. The adopted approach involved subtracting the 1954 water body allocation from the 1929 *unproductive area* category allocation. Although a few artificial canals or quarry lakes were likely established after the 1930s, the general configuration of both natural and human-made water bodies remained largely unchanged between the 1930s and 1950s [6]. To ensure consistency across the three periods, the *step_3.py* script was configured to permit an acceptable error margin of 5% when comparing the total area of the 1929 dataset with those of the two subsequent periods. The total area (ha) allocated to each LULC category across the three periods is summarised in Table 1 (see Results). Finally, the *step_3.py* script performs some exploratory data analysis (EDA) by generating two images: 1- pie charts illustrating LULC changes for the periods 1929, 1954, and 2024; 2- a Sankey diagram depicting LULC changes between 1954 and 2024 (Figs. 1 - 2 in Results). The latter EDA image requires a transition matrix calculated from raster data to assess pixel-level changes between the two periods. Since the 1929 LULC dataset is only available in vector format, with information aggregated at the administrative level (municipalities or provinces), it was not possible to include the 1929 LULC data in the Sankey diagram (Fig. 2 in Results).

C - Estimation of the agroforestry carbon pools

The AF carbon stock (CS) value was calculated using historical data from the 1929 Catasto, combined with allometric equations and MC simulations. The AF system considered in the calculation reflects the traditional *Coltura Promiscua* described in historical records. This system involved rows of mulberry trees with grapevines trained between them, interspersed with rows of crops, such as cereals (eg. *wheat*).

To estimate a realistic CS value, it was necessary to calculate the carbon pool for each of the four components of the AF system: Above-Ground Biomass (AGB), Below-Ground Biomass (BGB), Dead Matter (DM), and Soil Organic Carbon (SOC). The information retrieved from the 1929 Catasto provided a crucial foundation for these calculations. In particular, the key historical data included the mean number of mulberry trees per hectare (trees ha^{-1}), the mean wheat yield per hectare (t ha^{-1}), and the mean number of vine plants per hectare (plants ha^{-1}). This information was extracted from the GPKG vector layer developed in *step_1.py*, which summarised the 1929 Catasto data at the provincial scale.

To identify and exclude outliers from the historical dataset, a methodology based on the calculation of skewness was applied. Skewness, which measures the asymmetry of a data distribution, was used to determine the most appropriate statistical approach for outlier exclusion. The skewness of each dataset was calculated using an unbiased estimator to provide a reliable representation of the data distribution [38]. The choice of the outlier exclusion method was guided by the magnitude of the skewness value. When the absolute value of skewness was less than 1, indicating a symmetric or mildly skewed distribution, the trimmed mean method was applied. This approach removes a fixed percentage of the most extreme values from both ends of the distribution, reducing the influence of outliers while preserving the overall data structure. Conversely, when the absolute value of skewness was equal to or greater than 1, the dataset was classified as highly skewed. In such cases, the median was selected as the most robust central tendency measure, as it is less sensitive to the presence of extreme values compared to the mean. This systematic approach ensured the use of the most appropriate method for outlier exclusion based on the characteristics of the dataset, thereby improving

the reliability of the subsequent CS estimations. For the mulberry tree density (trees ha^{-1}) and the vine plants density (plants ha^{-1}) the skewness values are 1.05 and 2.53 respectively so the method chosen to exclude outliers is the median (skewness > 1) (Fig. S5 and S6). Conversely, for wheat yield rate (t ha^{-1}) the skewness value is <1 (0.17) and the selected method to exclude outliers is the trimmed mean (Fig. S7).

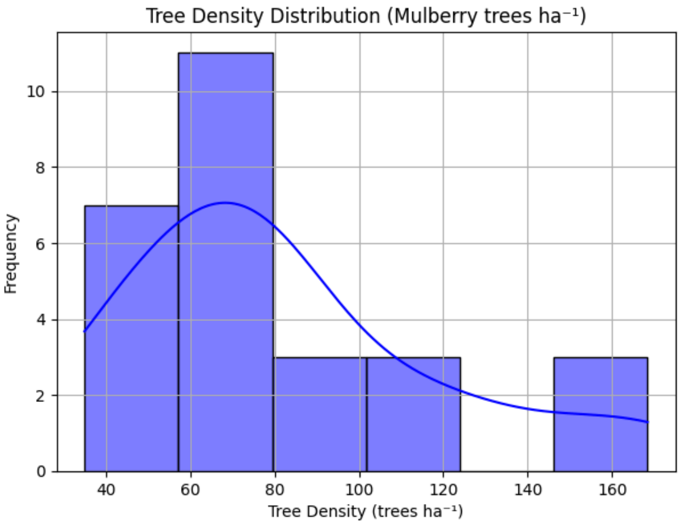

Fig. S5 - 1929 Mulberry tree density distribution (trees ha^{-1}). The distribution exhibits positive skewness, with a longer tail extending towards higher tree density values, suggesting a higher frequency of lower tree densities and a few instances of exceptionally high values. This skewness indicates the presence of potential outliers, particularly in the higher density range, which can disproportionately influence statistical measures like the mean. Consequently, a median-based method was applied to exclude these outliers, ensuring a more robust estimation of central tendency.

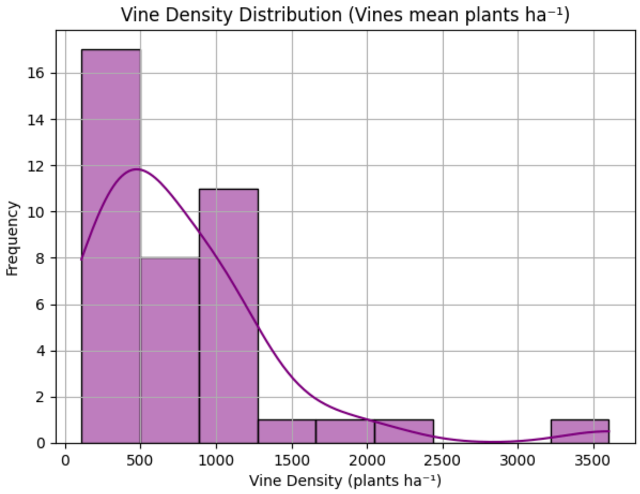

Fig. S6 - 1929 Vine plant density distribution (plants ha^{-1}). The distribution exhibits positive skewness, with a longer tail extending towards higher vine density values, indicating a higher frequency of lower density values and a few instances of exceptionally high values. This skewness suggests the presence of potential outliers, particularly in the higher density range, which can disproportionately influence statistical measures like the mean. Consequently, a median-based method was applied to exclude these outliers, ensuring a more robust estimation of central tendency.

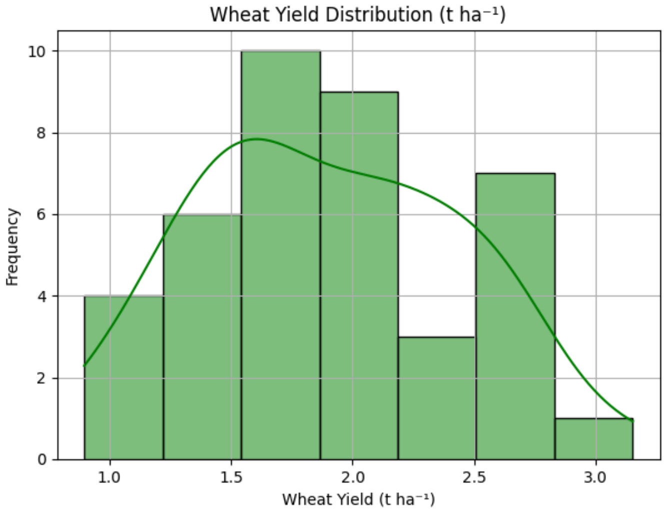

Fig. S7 - 1929 Wheat yield distribution (t ha^{-1}). The distribution appears relatively symmetric, with data points more evenly spread across the yield values. The absence of a pronounced skewness suggests a more balanced representation of wheat yields, with fewer extreme values. Given this near-symmetrical distribution, a trimmed mean method was applied to exclude potential outliers, ensuring a more reliable estimation of central tendency by minimising the influence of marginal extreme values.

To estimate the carbon pool (AGB, BGB, DM, SOC) for each component of the AF system (perennial: trees and vines; non-permanent: cereals), allometric equations were applied. Allometric equations are mathematical models that estimate vegetation biomass and carbon stock based on measurable variables such as diameter at breast height (DBH), plant height (PH), and wood density (WD). Derived from empirical data, these equations provide a non-destructive method for estimating biomass and carbon pools, making them essential for assessing carbon sequestration potential in AF systems [39,40].

The input values for these equations were obtained from field observations, historical records, scientific literature, and environmental agency reports. Given the high level of uncertainty associated with parameters such as SOC and the biological characteristics of trees at different growth stages, MC simulations were employed. This method simulates a broad range of possible values, thereby capturing variability and providing more robust and reliable CS estimates.

For mulberry trees (sp. *Morus alba*), AGB was estimated using the allometric equation (Eq. 1) derived from Mekonen et al. 2024 [41]:

$$(0.0559 * (WD * (DBH ** 2) * PH) ** 0.976)/1000000 \qquad \text{Eq. 1}$$

where:

- DBH: diameter at breast height (cm)
- PH: plant height (cm)
- WD: wood density (g/cm^3)

The parameter values used to estimate mulberry tree AGB were as follows: PH: 1–450 cm [12]; DBH: 1–60 cm [42]; WD: 0.670–0.850 g/cm^3 [43,44]. To account for variations in tree growth stages and the natural variability within the same species within the AF system, ranges of PH and DBH were incorporated into the MC simulations as well as the WD. A carbon fraction (CF) of 0.47 was applied to convert AGB into Above-Ground Carbon (AGC, measured in t C ha^{-1}). Below-Ground Biomass (BGB) was estimated from AGB using a root-to-shoot ratio between 0.12 and 1.21 [45] and subsequently converted into Below-Ground Carbon (BGC) stock using the same CF. The range of SOC values for the perennial component of the *Coltura Promiscua* (i.e. the mulberry trees) were retrieved from scientific literature focused on similar case studies that examined SOC in traditional AF systems across Europe: 0.219–0.617 t C $tree^{-1}$ [46–48]. The estimated AGC, BGC, and SOC for

individual plants were then multiplied by the range of tree densities, excluding outliers, resulting in a density range of 56 – 89 trees ha^{-1}.

For the non-permanent component (i.e. cereals), we calculated the corresponding carbon pool values using historical wheat yield data for 1929 [20]. The filtered data, originally reported in quintals per hectare, were converted to tonnes per hectare (t ha^{-1}) and ranged from 1.16 to 2.61 t ha^{-1}. The AGB of the non-permanent component was calculated using a harvest index (HI) of 0.5 [49], applying a modified equation (Eq. 2) from Gonçalves et al. (2024)[50]:

$$AGB = (Y \times (1 - HI)) / HI \quad \text{(Eq.2)}$$

where:

- Y: yield rate
- HI: harvest index

Since the land allocated to seasonal cultivation per hectare did not correspond to the total area, a more realistic approximation of the non-permanent component's contribution to the total AF carbon stock was achieved by calibrating the wheat AGB according to the hypothetical cropping area of *Coltura Promiscua*. This calibration was performed by estimating the area occupied by each tree, ranging between 10 and 30 m^2 (range estimated in QGIS observing the historic aerial images of *Volo Base Gruppo Aeronautico Italiano*), which represents the zone where cropping is limited (due to shading) or absent. Using the mean tree density per hectare mentioned previously, the maximum cropping area per hectare was determined through MC simulation.
Subsequently, a CF of 0.4 [51] was applied to the wheat AGB to derive the AGC, and the BGC stock was calculated using a root-to-shoot ratio ranging from 0.13 to 0.17 [52]. The SOC values for wheat were retrieved from the Italian Greenhouse Gas Inventory Report 2024 published by the Institute for Environmental Protection and Research (ISPRA) [53]. The values considered pertain to SOC in monoculture systems in Lombardy and Emilia-Romagna, considering all management types. The values used in this research, as reported in the ISPRA document, range from 26.95 to 65.19 t ha^{-1}.

For vine plants, carbon biomass values for a 10-year-old grapevine, including AGB, BGB, and DM components, were derived from Song et al. 2023 [54], where a single vine contributes 0.00118 t C. Following the same approach used for all other values in the calculation of AF CS, and to account for different stages of growth of individual vine plants, the MC simulation for the carbon pool of vines considered a range of values from a minimum of 0.00001 t C to the maximum value proposed by Song et al. 2023. The same source indicates that a single vine plant contributes 0.01583 t C to SOC. Following the same principle used for the vine biomass components, we considered all values between 0.00001 t C and the maximum reported by Song et al. 2023. Finally, these values for individual plants were multiplied by the filtered vine density range of 418.32 to 577.97 plants ha^{-1}.

To determine the appropriate number of simulations required for robust statistical inference, an initial sample of size (1000) was generated. This preliminary simulation aimed to estimate the standard deviation of the AF CS output by drawing values from uniform distributions defined for each input variable. The Central Limit Theorem (CLT) [55] was then applied to determine the required number of simulations to achieve a specified margin of error at a given confidence level. The CLT states that for a sufficiently large number of independent and identically distributed random samples, the distribution of the sample mean approaches a normal distribution, regardless of the shape of the original population distribution. This property is particularly useful in MC simulations where output values, such as AF CS, result from a combination of multiple stochastic processes. By leveraging the CLT, the number of required simulations (*n*) was calculated as:

$$n = \left(\frac{Z \cdot \sigma}{M}\right)^2$$

where *Z* is the critical value corresponding to the chosen confidence interval, σ is the estimated standard deviation of the initial sample, and specified margin of error (M).
Using the Central Limit Theorem (CLT) in this context provides two key advantages. First, optimized computational efficiency—rather than arbitrarily selecting many simulations, the CLT ensures that the number of iterations is sufficient to achieve statistical confidence. Second, reliable statistical

inference—since AF CS values are derived from stochastic parameters, their distribution may not be normal. However, the CLT guarantees that the distribution of sample means follows a normal distribution, enabling accurate confidence interval estimation. This approach allowed for an adaptive determination of the simulation size, ensuring a balance between computational efficiency and statistical accuracy.

The final MC simulations were conducted using parallel processing to efficiently utilize available CPU cores (Fig. S8). In each iteration, random samples were drawn from uniform probability distributions defined by parameter ranges for all the variables. The total AF CS was obtained by summing AGC, BGC, SOC contributions from the tree components of *Coltura Promiscua*. The DM component represents the transition of AGB to decomposing organic material, which contributes to long-term carbon storage in soil and litter pools. To account for variability in biomass turnover, the dead matter fraction was modelled using a uniform probability distribution in the range 10%–30%, This range reflects literature-reported decomposition rates in AF systems [56,57], capturing the uncertainty associated with species-specific and environmental differences in biomass decay rates.

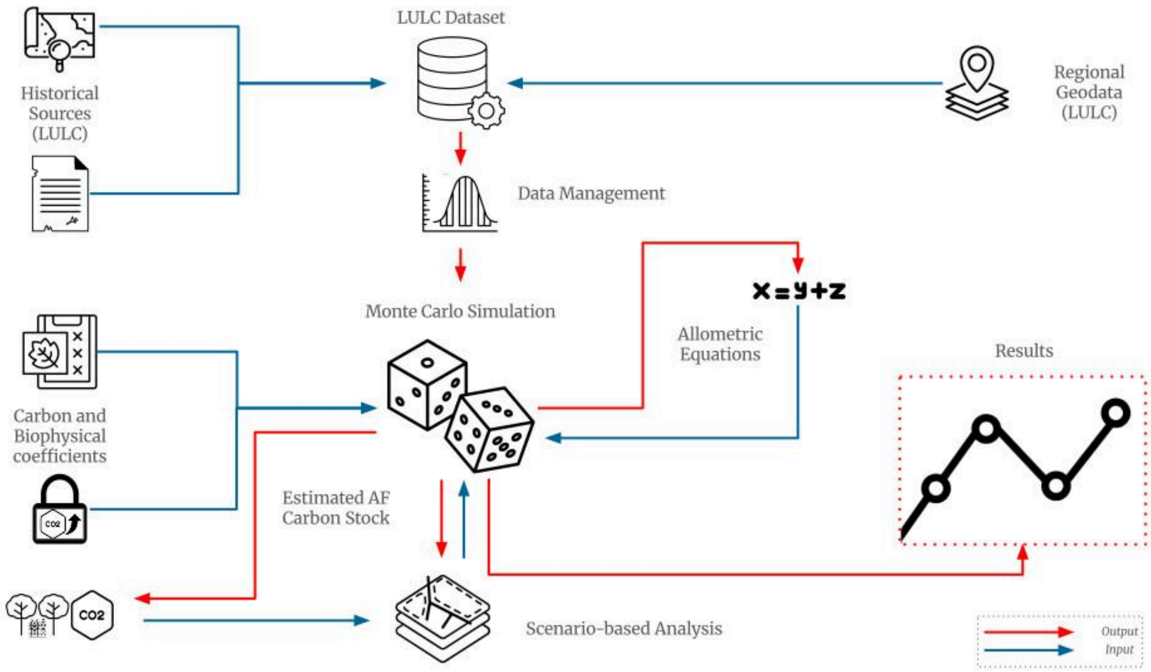

Fig. S8 - Methodological workflow adopted in this study. The diagram illustrates the integration of historical and regional land-use/land-cover (LULC) data with carbon and biophysical coefficients through a Monte Carlo simulation approach. Allometric equations and scenario-based analysis are used to estimate agroforestry (AF) carbon stock and generate results under varying assumptions. Blue arrows indicate data inputs; red arrows represent outputs.

References

1. Khomenko, S. *et al.* Premature mortality due to air pollution in European cities: a health impact assessment. *Lancet Planet. Health* **5**, e121–e134 (2021).
2. Castaldini, D., Marchetti, M., Norini, G., Vandelli, V. & Zuluaga Vélez, M. C. Geomorphology of the central Po Plain, Northern Italy. *J. Maps* **15**, 780–787 (2019).
3. Castiglioni G.B.; Pellegrini, G. B. *Note Illustrative Della Carta Geomorfologica Della Pianura Padana*. 328–421 (Supplementi di Geografia Fisica e Dinamica Quaternaria, Comitato Glaciologico Italiano, Torino, 2001).
4. Masetti, M. *et al.* Urbanization affects air and water in Italy's Po plain. *Eos* **96**, (2015).
5. European Commission. *Climate Action Progress Report 2024*. https://climate.ec.europa.eu/document/download/7bd19c68-b179-4f3f-af75-4e309ec0646f_en?filename=CAPR-report2024-web.pdf (2024).
6. Marchetti, M. Environmental changes in the central Po Plain (northern Italy) due to fluvial modifications and anthropogenic activities. *Geomorphology* **44**, 361–373 (2002).
7. Cremaschi, M. *et al.* Climate change versus land management in the Po Plain (Northern Italy) during the Bronze Age: New insights from the VP/VG sequence of the Terramara Santa Rosa di Poviglio. *Quat. Sci. Rev.* **136**, 153–172 (2016).
8. Mercuri, A. M. *et al.* Economy and environment of Bronze Age settlements – Terramaras – on the Po Plain (Northern Italy): first results from the archaeobotanical research at the Terramara di Montale. *Veg. Hist. Archaeobot.* **16**, 43–60 (2006).
9. Paris, P. *et al.* What is the future for agroforestry in Italy? *Agrofor. Syst.* **93**, 2243–2256 (2019).
10. Salvatico, A. *L'economia dell'alteno: viticoltura e cerealicoltura nel Roero e nelle Langhe tra il basso Medioevo e la prima età moderna*. (M. Valerio, 2004).
11. Ferrario, V. Aratorio arborato vitato. Il paesaggio agrario della coltura promiscua della vite tra fonti catastali e fonti cartografiche. in *Antico e sempre nuovo. L'agro centuriato a nord-est di Padova dall'Antichità all'Ottocento* (eds. Mengotti, C. & Bortolami, S.) 361–386 (Cierre Edizioni, Verona, 2012).
12. Sereni, E. *Storia Del Paesaggio Agrario Italiano*. (1961).

13. Bruno, L., Meli, M. & Garberi, M. L. Human-induced landscape modification in the last two centuries in the Po delta plain (Northern Italy). *Anthropocene* **48**, 100453 (2024).
14. Schönafinger, A., Egarter Vigl, L. & Tasser, E. Spatiotemporal patterns and drivers of orchard meadow loss in South Tyrol, Italy. *Sci. Rep.* **14**, 30812 (2024).
15. Ferrario, V. Learning from Agricultural Heritage? Lessons of Sustainability from Italian 'Coltura Promiscua'. *Sustain. Sci. Pract. Policy* **13**, 8879 (2021).
16. Steffen, W., Broadgate, W., Deutsch, L., Gaffney, O. & Ludwig, C. The trajectory of the Anthropocene: The Great Acceleration. *Anthr. Rev.* **2**, 81–98 (2015).
17. Bonanno, A. Theories of the State: The Case of Land Reform in Italy, 1944-1961. *The Sociological Quarterly* **29**, 131–147 (1988).
18. Romano, B., Zullo, F., Fiorini, L., Marucci, A. & Ciabò, S. Land transformation of Italy due to half a century of urbanization. *Land use policy* **67**, 387–400 (2017).
19. Smiraglia, D., Ceccarelli, T., Bajocco, S., Perini, L. & Salvati, L. Unraveling landscape complexity: Land use/land cover changes and landscape pattern dynamics (1954-2008) in contrasting Peri-urban and Agro-forest regions of northern Italy. *Environ. Manage.* **56**, 916–932 (2015).
20. ISTAT. Catasto Agrario 1929. https://lipari.istat.it/digibib/Catasto/Catasto%20agrario%201929/ (1929).
21. ABBYY. *FineReader PDF 16*. https://help.abbyy.com/assets/en-us/finereader/16/Users_Guide.pdf (2023).
22. QGIS Development Team. *QGIS Geographic Information System. Open Source Geospatial Foundation Project*. (2025).
23. Lieskovský, J. *et al. Historical Land Use Dataset of the Carpathian Region (1819-1980)*. (2018).
24. Brandolini, F., Reynard, E. & Pelfini, M. Multi-temporal mapping of the Upper Rhone Valley (Valais, Switzerland): fluvial landscape changes at the end of the Little Ice Age (18th–19th centuries). *J. Maps* **16**, 212–221 (2020).
25. Oniga, V.-E., Breaban, A.-I. & Statescu, F. Determining the Optimum Number of Ground Control Points for Obtaining High Precision Results Based on UAS Images. *Proc. AMIA Annu.*

Fall Symp. **2**, 352 (2018).

26. Baiocchi, V., Lelo, K., Milone, M. V. & Mormile, M. Accuracy of different georeferencing strategies on historical maps of Rome. *Geographia Technica* **1**, 10–16 (2013).
27. Nazionale, G. Geoportale Nazionale. *Geoportale Nazionale* http://www.pcn.minambiente.it/mattm/ (2024).
28. Yutzler, J. & Daise, P. *OGC® - GeoPackage Encoding Standard*. (2021).
29. The pandas development team. *Pandas-Dev/pandas: Pandas*. (Zenodo, 2024). doi:10.5281/ZENODO.3509134.
30. Jordahl, K. *et al. Geopandas/geopandas: v0.8.1*. (Zenodo, 2020). doi:10.5281/zenodo.3946761.
31. GRASS Development Team. *Geographic Resources Analysis Support System (GRASS)*. (Open Source Geospatial Foundation, 2023).
32. European Environment Agency. *The Revised and Supplemented CORINE Land Cover Nomenclature: Updated CLC Illustrated Nomenclature Guidelines*. https://land.copernicus.eu/content/corine-land-cover-nomenclature-guidelines/docs/pdf/CLC2018_Nomenclature_illustrated_guide_20190510.pdf (2019).
33. Lombardia, G. Geoportale Lombardia. *Regione Lombardia* https://www.geoportale.regione.lombardia.it/ (2025).
34. Geoportale, E. R. Geoportale Emilia Romagna. *Regione Emilia Romagna* https://geoportale.regione.emilia-romagna.it/ (2025).
35. Van Den Berge, S. *et al.* Soil carbon of hedgerows and 'ghost' hedgerows. *Agrofor. Syst.* **95**, 1087–1103 (2021).
36. Drexler, S., Gensior, A. & Don, A. Carbon sequestration in hedgerow biomass and soil in the temperate climate zone. *Reg. Environ. Change* **21**, 1–14 (2021).
37. Drexler, S., Thiessen, E. & Don, A. Carbon storage in old hedgerows: The importance of below‐ground biomass. *Glob. Change Biol. Bioenergy* **16**, (2024).
38. Sirkin, R. M. *Statistics for the Social Sciences*. 632 (SAGE Publications, Inc, Thousand Oaks, CA, 2005).
39. Andrade, H. J., Segura, M. A. & Feria, M. Allometric models for estimating belowground

biomass of individual coffee bushes growing in monoculture and agroforestry systems. *Agrofor. Syst.* **95**, 215–226 (2021).

40. Schindler, Z., Morhart, C., Sheppard, J. P., Frey, J. & Seifert, T. In a nutshell: exploring single tree parameters and above-ground carbon sequestration potential of common walnut (Juglans regia L.) in agroforestry systems. *Agrofor. Syst.* **97**, 1007–1024 (2023).
41. Mekonen, A. A., Accardo, D. & Renga, A. Above Ground Biomass Estimation in Agroforestry Environment by UAS and RGB Imagery. in *2024 11th International Workshop on Metrology for AeroSpace (MetroAeroSpace)* 272–277 (IEEE, 2024).
42. Brandolini, F., Pelfini, M. & Turner, S. The Evolution of Historic Agroforestry Landscape in the Northern Apennines (Italy) and Its Consequences for Slope Geomorphic Processes. *Land* **12**, 1054 (2023).
43. Reyes, G., Brown, S., Chapman, J. & Lugo, A. E. *Wood Densities of Tree Species*. http://dx.doi.org/10.2737/so-gtr-88 (1992) doi:10.2737/so-gtr-88.
44. Orwa, C., Mutua, A., Kindt, R., Jamnadass, R. & Anthony, S. *Morus Alba*. (Agroforestry Database 4.0, World Agroforestry Centre (ICRAF), 2009).
45. Levy, P. E., Hale, S. E. & Nicoll, B. C. Biomass expansion factors and root : shoot ratios for coniferous tree species in Great Britain. *Forestry* **77**, 421–430 (2004).
46. Navarro-Rosales, F., Fernández-Habas, J., Reyna-Bowen, L., Gómez, J. A. & Fernández-Rebollo, P. Subsoiling for planting trees in dehesa system: long-term effects on soil organic carbon. *Agrofor. Syst.* **97**, 699–710 (2023).
47. Ramananjatovo, T. *et al.* Positive influence of apple trees on soil chemical and biological activities in agroecological garden orchard system. *Agrofor. Syst.* **98**, 3233–3246 (2024).
48. Fornara, D. A. *et al.* Land use change and soil carbon pools: evidence from a long-term silvopastoral experiment. *Agrofor. Syst.* **92**, 1035–1046 (2018).
49. Chen, J. *et al.* Diversity increases yield but reduces harvest index in crop mixtures. *Nat. Plants* **7**, 893–898 (2021).
50. Gonçalves, R., Wamelink, G. W. W., van der Putten, P. & Evers, J. B. Intercropping on Mars: A promising system to optimise fresh food production in future martian colonies. *PLoS One* **19**,

e0302149 (2024).

51. Bolinder, M. A., Angers, D. A. & Dubuc, J. P. Estimating shoot to root ratios and annual carbon inputs in soils for cereal crops. *Agric. Ecosyst. Environ.* **63**, 61–66 (1997).

52. Williams, J. D. *et al.* Root:shoot ratios and belowground biomass distribution for Pacific Northwest dryland crops. *J. Soil Water Conserv.* **68**, 349–360 (2013).

53. Romano, D. *et al. Italian Greenhouse Gas Inventory 1990-2022. National Inventory Report 2024*. vol. 398 https://www.isprambiente.gov.it/files2024/pubblicazioni/rapporti/nir-2024-r-398-24.pdf (2024).

54. Song, R., Zhu, Z., Zhang, L., Li, H. & Wang, H. A Simple Method Using an Allometric Model to Quantify the Carbon Sequestration Capacity in Vineyards. *Plants* **12**, (2023).

55. Central Limit Theorem. in *The Concise Encyclopedia of Statistics* 66–68 (Springer New York, New York, NY, 2008).

56. Ramachandran Nair, P. K., Mohan Kumar, B. & Nair, V. D. *An Introduction to Agroforestry*. (Springer International Publishing, 2021).

57. Eggleston, H. S., Buendia, L., Miwa, K., Ngara, T. & (Eds.), K. T. *2006 IPCC Guidelines for National Greenhouse Gas Inventories, Volume 4: Agriculture, Forestry and Other Land Use*. (Institute for Global Environmental Strategies (IGES) on behalf of the IPCC, Hayama, Japan, 2006).
